# Extracellular matrix proteomics identifies molecular signature of symptomatic carotid plaques

**DOI:** 10.1172/JCI86924

**Published:** 2017-03-20

**Authors:** Sarah R. Langley, Karin Willeit, Athanasios Didangelos, Ljubica Perisic Matic, Philipp Skroblin, Javier Barallobre-Barreiro, Mariette Lengquist, Gregor Rungger, Alexander Kapustin, Ludmilla Kedenko, Chris Molenaar, Ruifang Lu, Temo Barwari, Gonca Suna, Xiaoke Yin, Bernhard Iglseder, Bernhard Paulweber, Peter Willeit, Joseph Shalhoub, Gerard Pasterkamp, Alun H. Davies, Claudia Monaco, Ulf Hedin, Catherine M. Shanahan, Johann Willeit, Stefan Kiechl, Manuel Mayr

**Affiliations:** 1 King’s British Heart Foundation Centre, King’s College London, London, United Kingdom.; 2 Duke-NUS Medical School, Singapore.; 3 Department of Neurology, Medical University Innsbruck, Innsbruck, Austria.; 4 Department of Molecular Medicine and Surgery, Vascular Surgery, Karolinska Institute, Stockholm, Sweden.; 5 Department of Neurology, Bruneck Hospital, Bruneck, Italy.; 6 First Department of Internal Medicine, Paracelsus Medical University, Salzburg, Austria.; 7 Nikon Imaging Centre, King’s College London, London, United Kingdom.; 8 Department of Geriatric Medicine, Paracelsus Medical University, Salzburg, Austria.; 9 Department of Public Health and Primary Care, University of Cambridge, Cambridge, United Kingdom.; 10 Department of Surgery and Cancer, Imperial College London, London, United Kingdom.; 11 Laboratory of Clinical Chemistry and Experimental Cardiology, University Medical Center Utrecht, Utrecht, The Netherlands.; 12 Kennedy Institute, University of Oxford, Oxford, United Kingdom

## Abstract

**BACKGROUND.** The identification of patients with high-risk atherosclerotic plaques prior to the manifestation of clinical events remains challenging. Recent findings question histology- and imaging-based definitions of the “vulnerable plaque,” necessitating an improved approach for predicting onset of symptoms.

**METHODS.** We performed a proteomics comparison of the vascular extracellular matrix and associated molecules in human carotid endarterectomy specimens from 6 symptomatic versus 6 asymptomatic patients to identify a protein signature for high-risk atherosclerotic plaques. Proteomics data were integrated with gene expression profiling of 121 carotid endarterectomies and an analysis of protein secretion by lipid-loaded human vascular smooth muscle cells. Finally, epidemiological validation of candidate biomarkers was performed in two community-based studies.

**RESULTS.** Proteomics and at least one of the other two approaches identified a molecular signature of plaques from symptomatic patients that comprised matrix metalloproteinase 9, chitinase 3-like-1, S100 calcium binding protein A8 (S100A8), S100A9, cathepsin B, fibronectin, and galectin-3-binding protein. Biomarker candidates measured in 685 subjects in the Bruneck study were associated with progression to advanced atherosclerosis and incidence of cardiovascular disease over a 10-year follow-up period. A 4-biomarker signature (matrix metalloproteinase 9, S100A8/S100A9, cathepsin D, and galectin-3-binding protein) improved risk prediction and was successfully replicated in an independent cohort, the SAPHIR study.

**CONCLUSION.** The identified 4-biomarker signature may improve risk prediction and diagnostics for the management of cardiovascular disease. Further, our study highlights the strength of tissue-based proteomics for biomarker discovery.

**FUNDING.** UK: British Heart Foundation (BHF); King’s BHF Center; and the National Institute for Health Research Biomedical Research Center based at Guy’s and St Thomas’ NHS Foundation Trust and King’s College London in partnership with King’s College Hospital. Austria: Federal Ministry for Transport, Innovation and Technology (BMVIT); Federal Ministry of Science, Research and Economy (BMWFW); Wirtschaftsagentur Wien; and Standortagentur Tirol.

## Introduction

Atherosclerosis is a chronic and progressive disease of the arterial wall and the main underlying cause of stroke, myocardial infarction (MI), and cardiac death (1, 2). The evolution of atherosclerotic plaques involves endothelial dysfunction, accumulation of lipids and inflammatory cells, as well as remodeling of the extracellular matrix (ECM). Previously, atherosclerotic plaques were defined by histological appearance. Plaques with thin fibrous caps and a large lipid pool were classified as “vulnerable lesions.” Lipid-poor plaques rich in ECM with thick fibrous caps were considered “stable.” Recent findings have challenged this “vulnerable plaque” concept (3, 4): First, intravascular imaging revealed that only a small percentage of thin-capped plaques cause clinical events (4, 5). Second, shifts in risk factor profiles (e.g., smoking cessation) and widespread use of statins are associated with a change in histopathological appearance of atherosclerotic lesions: “vulnerable plaques” with large lipid pools are far less common (3). Instead, superficial plaque erosions may trigger more cardiovascular events (3, 4).

Since histological appearance alone is insufficient to predict symptomatic disease, new approaches are needed to define the biological features of atherosclerotic lesions responsible for cardiovascular syndromes and to test whether such features could be of clinical value in predicting events (4). Markers of systemic inflammation such as C-reactive protein (CRP) are elevated in subjects at risk for cardiovascular disease (CVD) but lack the specificity required for reliable differentiation between lesions from symptomatic and asymptomatic patients (6). Recent advances in elucidating molecular processes involved in plaque destabilization have highlighted the importance of the vascular ECM and its associated proteins (7, 8). We have previously developed a proteomics methodology allowing a detailed analysis of the vascular ECM and associated proteins in the extracellular space (9, 10).

The aim of the present study was to identify a protein signature for symptomatic plaques by applying a tissue- and cell-based proteomics discovery approach involving carotid endarterectomy specimens and secretome analysis of lipid-loaded human vascular smooth muscle cells (SMCs). The proteomics data were integrated with plaque transcriptomic data from the Biobank of Karolinska Endarterectomies (BiKE) (11). The final selection of candidate biomarkers and a 4-biomarker signature derived from the individual components was validated in the community-based Bruneck study, which is unique in its long-term follow-up and detailed monitoring of atherosclerosis manifestation and progression, and in a second independent cohort (Salzburg Atherosclerosis Prevention program in subjects at High Individual Risk [SAPHIR] study).

## Results

### Proteomics of the ECM of carotid endarterectomies.

We applied our published three-step extraction methodology (9, 10) to analyze ECM and ECM-associated proteins of carotid endarterectomy specimens previously analyzed by lipidomics (12). Six were from symptomatic and 6 from asymptomatic patients, with equal age and sex distribution. To enhance classification accuracy, we selected symptomatic patients who had ipsilateral carotid territory established stroke documented by CT or MRI, while transient ischemic attack or amaurosis fugax relying on the patient self-report only were not considered. The patient characteristics are summarized in Supplemental Table 1 (supplemental material available online with this article; https://doi.org/10.1172/JCI86924DS1). Tissue decellularization was performed by 0.08% SDS to reduce the highly abundant cellular proteins (9, 10). The two remaining fractions were analyzed by proteomics using a high mass accuracy tandem mass spectrometer: the initial salt (0.5 M NaCl) fraction, which contains newly synthesized and degraded extracellular proteins, and the final guanidine (4 M GuHCl) fraction, which contains predominantly ECM proteins and proteins tightly associated with the ECM in the vasculature (9, 10). Using a 95% peptide probability, 99% protein probability, 10 ppm mass accuracy, a minimum of two peptides per protein and detection in at least 2 samples in each group, we found 110 ECM or ECM-associated proteins in the GuHCl fraction and 87 in the NaCl fraction, with an overlap of 51 (Supplemental Table 2).

**Figure 1:**
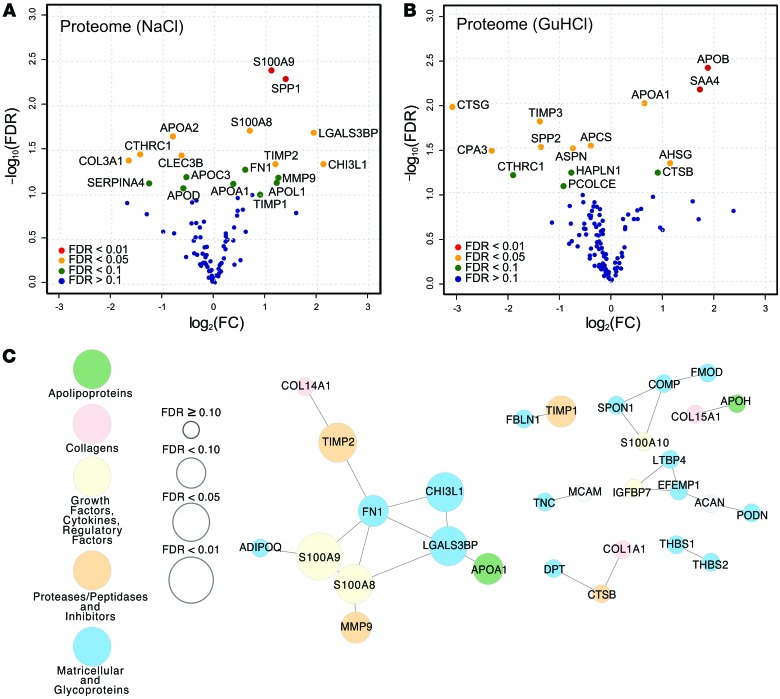
ECM proteomics of carotid endarterectomies. Volcano plots of differences in abundance in (**A**) the proteome of the NaCl fraction (*n* = 12) and (**B**) the proteome of the GuHCl fraction (*n* = 12) between symptomatic and asymptomatic patients. Colors represent FDR levels (red, FDR <1%; orange, FDR <5%; green, FDR <10%; blue, FDR ≥10%, by NSAF-PLGEM; FC, fold change), and the labeled dots are those that were significantly differentially expressed between the plaques from symptomatic and asymptomatic patients (FDR <10%). (**C**) Coexpression network in the NaCl fraction (*n* = 12) calculated from Pearson correlations with edges defined at FDR <20% (*Q* values). The sizes of the nodes indicate the level of confidence for the differential expression between plaques from symptomatic and asymptomatic patients, and the colors represent different protein classifications (yellow, growth factors, cytokines, and regulatory factors; green, apolipoproteins; pink, collagens; orange, proteases/peptidases and inhibitors; blue, matricellular proteins and glycoproteins; white, other).

When comparing carotid plaques from symptomatic and asymptomatic patients, we identified 18 proteins in the NaCl (Figure 1A) and 14 in the GuHCl fraction (Figure 1B) that were differentially expressed with an FDR <10%. Collagen triple helix repeat–containing protein 1 (CTHRC1) and apolipoprotein A1 (APOA1) proteins were identified in both fractions (Table 1). According to a disease association analysis, the 30 differentially expressed proteins were enriched for several disease terms, including fibrosis, arterial occlusive diseases, vascular disease, and arteriosclerosis (FDR <0.001; WEB-based GEne SeT AnaLysis Toolkit; http://www.webgestalt.org). Protein coexpression networks for the NaCl and the GuHCl fractions are shown in Figure 1C and Supplemental Figure 1, respectively. Characterization of classical cellular markers and immunoblot confirmation of differences for selected proteins are shown in Supplemental Figure 2. Plaques from symptomatic patients had less smooth muscle actin (SMA), but more osteopontin (SPP1), matrix metalloproteinase 9 (MMP9), and fibronectin (FN1) (Supplemental Figure 3).

### Transcriptomic profiling of carotid endarterectomies.

**Figure 2:**
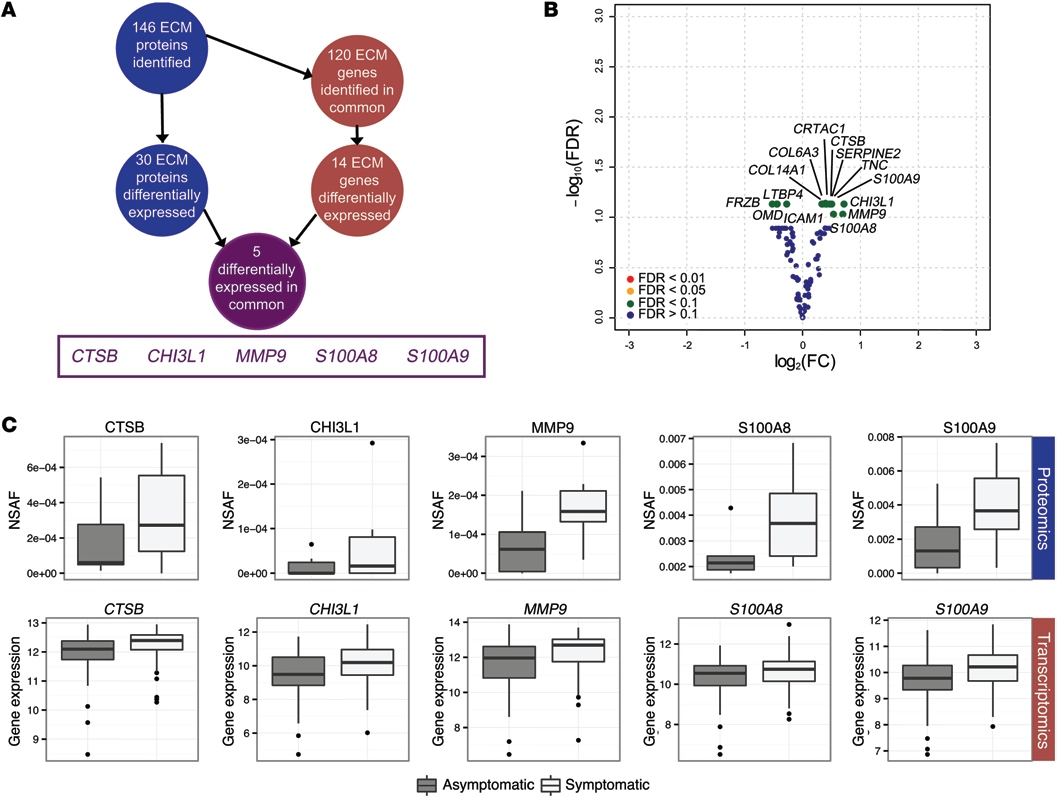
Comparison between protein and gene expression levels. (**A**) Schematic for detecting differentially expressed proteins that were also differentially expressed at the mRNA transcript level. (**B**) Volcano plot of differences in expression between symptomatic and asymptomatic patients (*n* = 121) for corresponding ECM-associated genes (green, FDR <10%; blue, FDR ≥10%, by limma), and the labeled dots are those that were significantly differentially expressed between the plaques from symptomatic and asymptomatic patients (FDR <10%). (**C**) Box plots for the 5 molecules in common for protein abundance (NSAF) and normalized gene expression (*n* = 12, proteomics; *n* = 121, transcriptomics; FDR <10%). The box plots are as follows: black line, median; box edges, 1st and 3rd quartiles; whiskers, furthest point within 1.5 times the interquartile range.

In order to validate our proteomics findings, we interrogated the corresponding gene expression data for ECM and ECM-associated proteins in 121 carotid plaques of the BiKE cohort (from 84 symptomatic and 37 asymptomatic patients; Figure 2A) (11). With this approach, 14 transcripts (Figure 2B) were found to be differentially expressed between plaques from symptomatic and asymptomatic patients, with an FDR <10% (Table 2). Of the 30 differentially expressed proteins (ECM proteomics; Figure 1) and 14 differentially expressed genes (ECM transcriptomics, Figure 2B), 5 were altered at both the transcript and the protein level (Figure 2C): cathepsin B (*CTSB*), chitinase 3-like-1 (*CHI3L1*), matrix metalloproteinase 9 (*MMP9*), S100 calcium binding protein A8 (*S100A8*), and *S100A9*. All of these genes and corresponding proteins were positively correlated (Supplemental Figure 4) and upregulated in plaques from symptomatic versus asymptomatic patients.

### Secretome analysis of lipid-loaded human SMCs.

**Figure 3:**
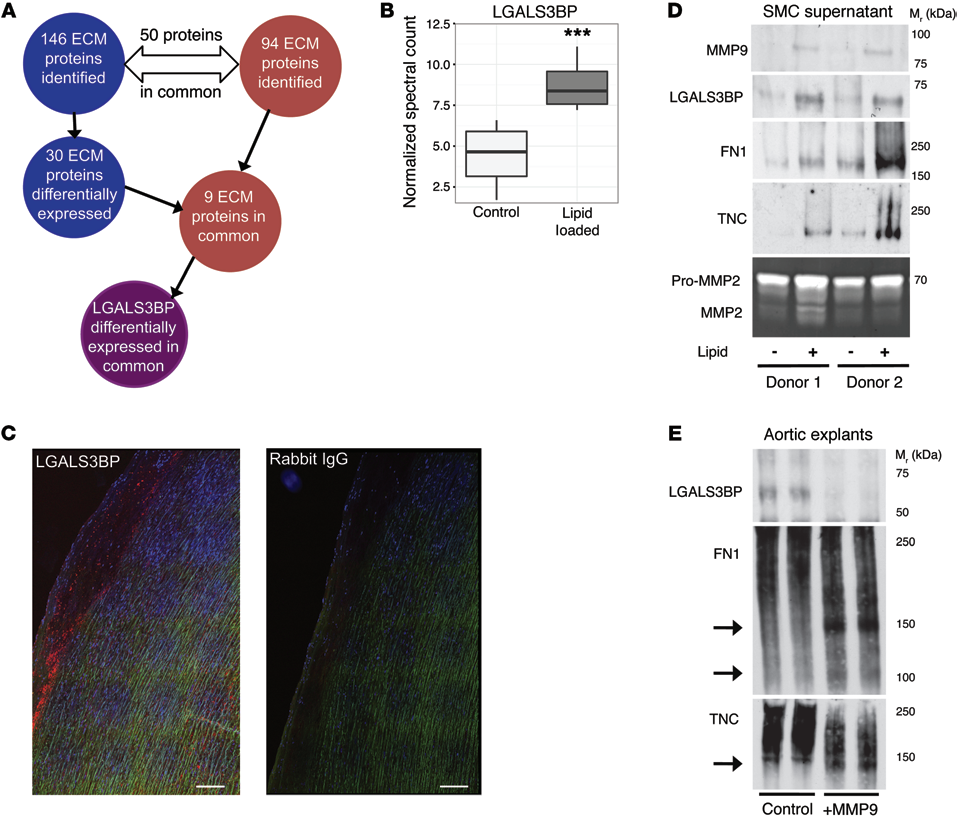
Proteomics of the secretome from lipid-loaded human vascular SMCs. (**A**) Schematic for detecting differentially expressed ECM proteins that also showed differential secretion in lipid-loaded human vascular SMCs. (**B**) The proteomics analysis of the conditioned media (“secretome”) showed the LGALS3BP level to be significantly higher in the lipid-loaded versus control SMCs (*n* = 6, ***P = 0.005, paired *t* test). The box plot is as follows: black line, median; box edges, 1st and 3rd quartile; whiskers, furthest point within 1.5 times the interquartile range. (**C**) Immunofluorescence staining for LGALS3BP (red) in early atherosclerosis (human ascending aorta). Autofluorescence of elastin fibers (green). Scale bars: 200 μm. (**D**) Validation by immunoblotting. Note the co-detection of MMP9, FN1, LGALS3BP, and TNC upon lipid loading. SMCs from two different donors. Gelatin zymography for MMP2 was used as loading control. (**E**) Human aortic explants were either incubated with 50 pM MMP9 or left untreated for 24 hours. The degradation of FN1, TNC, and LGALS3B was examined by immunoblotting. MMP9 induces degradation of FN1 and TNC but releases LGALS3BP from human aortic explants. Aortic explants were from 2 different donors. Arrows indicate fragmentation products.

Recent findings using lineage tracing demonstrated that the contribution of SMCs to atherosclerotic plaques and to the regulation of proinflammatory responses has been greatly underestimated (13). Thus, we complemented our tissue-based proteomics approach by a proteomics analysis of protein secretion by vascular SMCs. Human vascular SMCs were converted to a foam-like atherosclerotic phenotype using lipid loading as previously reported (14). Proteomics analyses were then performed on the ECM deposited by the cell layer and the conditioned media (Supplemental Tables 3 and 4). Approximately 50% of the ECM proteins identified in lipid-loaded SMCs were in common with those in human atherosclerotic plaques, highlighting the importance of SMCs to pathological ECM changes in the vessel wall (Figure 3A). Galectin-3-binding protein (LGALS3BP) secretion was markedly increased in the conditioned media of lipid-loaded SMCs (*P* = 0.005, Figure 3B and Supplemental Table 4) and was observed in early atherosclerotic lesions in the human ascending aorta (Figure 3C). LGALS3BP was also differentially expressed in plaques from symptomatic versus asymptomatic patients (Figure 1A, FDR = 0.02); connected with MMP9 and FN1 in the protein coexpression network (Figure 1C); and showed co-detection with MMP9, FN1, and tenascin C (TNC) in the conditioned medium of lipid-loaded SMCs (Figure 3D). Furthermore, when human aortic explants were treated with recombinant MMP9 ex vivo, proteolytic cleavage of FN1 and TNC co-occurred with a release of LGALS3BP from the vascular ECM (Figure 3E).

### Immunohistological staining in carotid plaques.

**Figure 4:**
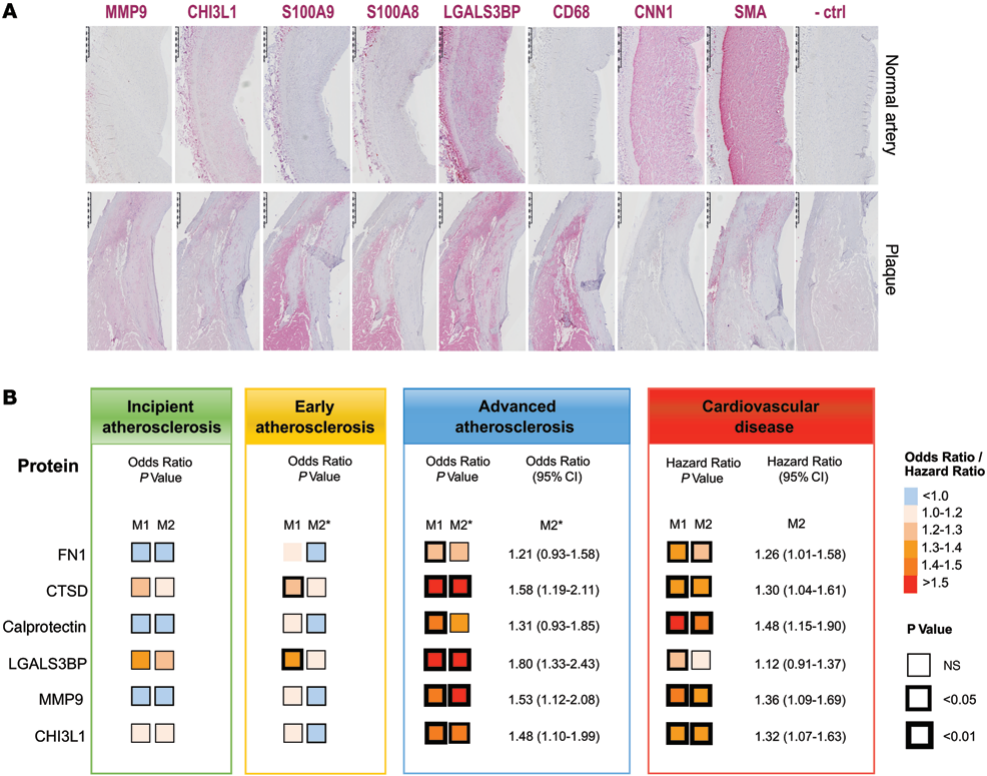
Biomarker candidates in tissue and plasma. (**A**) Immunohistochemistry staining for LGALS3BP, S100A8/A9, CHI3L1, and MMP9 plus SMC (SMA, CNN1) and macrophage (CD68) markers. Scale bars: 500 μm. (**B**) Association between the biomarker candidates and atherosclerosis (*n* = 560) and CVD (*n* = 685) in the Bruneck study. Direction and strength of associations are coded by color and significance levels by size of frames. ORs are derived from logistic regression analysis, and hazard ratios from Cox models. ORs and hazard ratios are expressed for a 1-SD higher log_e_-transformed level of the given marker. Full details are presented in Supplemental Tables 6 and 7. Model 1 (M1) was adjusted for age (years) and sex. M2 was additionally controlled for standard vascular risk factors, including LDL cholesterol (mg/dl), HDL cholesterol (mg/dl), high-sensitivity CRP (mg/l), diabetes mellitus (0 vs. 1), hypertension (0 vs. 1), smoking (pack-years), and body mass index (kg/m^2^). *Additionally adjusted for atherosclerosis score (mm).

Immunohistological staining for the identified proteins (S100A8, S100A9, CHI3L1, MMP9, LGALS3BP) along with cell markers (CD68: macrophage marker, CNN and SMA: SMC markers) was performed on consecutive sections. Representative images from normal artery and carotid plaques are shown in Figure 4A. S100A8 and S100A9 localized in the macrophage-rich areas in plaques, as shown by the CD68 staining, and were largely absent in normal arteries. Staining for MMP9 was observed throughout the plaque and was also absent in the control. CHI3L1 and LGALS3BP were present in both the normal and diseased artery but stronger in plaques. The latter is consistent with the detection of LGALS3BP in SMC cultures.

### Epidemiological validation in two community-based studies.

Baseline characteristics of subjects in the Bruneck study are shown in Supplemental Table 5. Mean age was 66.1 years, and 355 (51.8%) were female. During the 10-year follow-up 91 of the 685 individuals experienced the composite CVD end point of MI, stroke, or vascular death. A total of 560 individuals had ultrasonographic follow-up, with 102 showing progression to advanced complicated stages of plaque development (advanced atherogenesis) and 371 developing new atherosclerotic plaques or exhibiting progression of non-stenotic plaques (early atherogenesis), including 129 of 278 subjects without carotid atherosclerosis at baseline.

Our discovery approach identified the following final selection of candidate proteins as an overlap of ECM proteomics with either ECM transcriptomics or SMC secretome analysis: MMP9, CHI3L1, S100A8, S100A9, CTSB, FN1, and LGALS3BP (Supplemental Figure 5). In the Bruneck Study, baseline plasma levels of FN1 and cathepsin D (CTSD) were available as part of two biomarker panels measured by proximity extension assays (CVD I and Inflammation I Panel, Olink) (15). Both CTSD and CTSB are lysosomal cathepsins, and CTSD was used as a surrogate of CTSB due to their similar localization and high inter-correlation. Dimers of S100A8 and S100A9 form the inflammatory protein complex calprotectin. Calprotectin, CHI3L1, MMP9, and LGALS3BP were measured by ELISA.

Baseline levels of these candidate biomarkers showed significant associations with advanced atherogenesis (under adjustment for age and sex), whereas no or weak associations existed for early atherogenesis (Figure 4B and Supplemental Tables 6 and 7). Moreover, all these proteins emerged as predictive of CVD events during the 10-year follow-up (age- and sex-adjusted model). Most associations remained robust in multivariable models. In line with our previous analyses (16), the standard vascular risk factors of high LDL cholesterol, hypertension, and cumulative exposure to smoking (pack-years) significantly related to early but not advanced atherogenesis. Several of the identified markers of symptomatic plaques provide distinct and additive information. A final risk model, built by a forward stepwise selection procedure and allowing for all biomarkers, comprised CTSD (odds ratio [OR] [95% CI], 1.3 [1.0–1.6]), LGALS3BP (OR [95% CI], 1.5 [1.1–1.9]), MMP9 (OR [95% CI], 1.3 [1.0–1.6]), and calprotectin (OR [95% CI] 1.3 [1.0–1.7]) for the ultrasound end point (advanced atherogenesis). In this model, CTSD could be exchanged by CHI3L1, both of which were highly correlated (*r* = 0.51). The 4-biomarker signature unraveled for advanced atherogenesis also performed well for incident CVD.

Additionally, in a series of patients with recently symptomatic carotid stenosis (*n* = 17), we measured plasma levels of MMP9 and calprotectin and found them to be 2.2 and 1.6 times higher (fold change of geometric means) than in individuals from the Bruneck study (free of CVD and carotid stenosis, *n* = 516) under adjustment for age and sex (*P* < 0.001 each).

Next, we strove for additional replication in an independent cohort — the SAPHIR study (baseline characteristics shown in Supplemental Table 8). We chose a case-cohort design and identified 58 cases of incident CVD during a median follow-up of 11.9 (interquartile range, 10.3–13.2) years. Four of the 5 biomarkers measured in SAPHIR showed associations with incident CVD, while replication failed for one (CHI3L1; Supplemental Figure 6). As to the latter finding, it has to be considered that the SAPHIR cohort was 14.3 years younger on average, and composition of the CVD end point differed in Bruneck (stroke > MI) and SAPHIR (MI > stroke). This may be relevant for CHI3L1 because previous studies have reported an exclusive association with stroke (17).

In the Bruneck study, consideration of the 4-biomarker signature along with standard CVD risk factors improved the C-statistic for the risk of advanced atherosclerosis by 0.048 (*P* = 0.0038), surpassing the improvement that was gained considering its individual components separately (Supplemental Figure 7). Furthermore, consideration of the 4-biomarker signature significantly improved CVD risk prediction in terms of discrimination and risk classification (Supplemental Figure 8). Finally, the incremental predictive value of the same 4-biomarker panel was similar in the SAPHIR study (Supplemental Figure 8).

## Discussion

To date it is still not possible to accurately identify patients with high-risk atherosclerotic plaques before the manifestation of clinical events. Here, we used three different approaches to discover a molecular signature for plaque instability that does not rely on imaging or histopathological parameters but can be measured in the circulation: First, a novel proteomics methodology targeting the ECM and ECM-associated proteins was applied to 12 carotid endarterectomy specimens (from symptomatic versus asymptomatic patients), and selected findings were confirmed by immunoblotting. Second, the proteomics findings were integrated with the corresponding ECM-associated transcriptomic profiles of 121 carotid endarterectomies from the BiKE cohort (11). Third, an in-depth analysis of proteins secreted by lipid-loaded versus unstimulated human SMCs (secretome) was performed. Proteins were considered candidate biomarkers for plaque destabilization when detected by plaque proteomics and at least one of the two other approaches. Epidemiological validation of the candidate biomarkers was performed in two independent prospective studies — the Bruneck and SAPHIR studies — and a final 4-biomarker signature was identified that significantly improved clinical risk prediction along with the information provided by standard CVD risk factors (Supplemental Figure 8).

The vascular ECM is crucial for the structural organization and integrity of the artery wall (7, 8). The ECM provides a scaffold for attachment of molecules secreted by vascular cells, as well as for molecules entering from the circulation. In disease states, degradation and pathological remodeling of the ECM promotes plaque development and progression by trapping circulating lipoproteins and growth factors, which exert biological activity and critically determine plaque stability. To our knowledge, the current study is the first comprehensive proteomics analysis of the ECM in human atherosclerotic plaques, whereas previous studies have focused on the cellular proteome (18) or particular components of the ECM (proteoglycans) (19). Our study provides insights into the composition of advanced atherosclerotic lesions featured by high levels of MMP9, CHI3L1, S100A8-S100A9 (calprotectin), lysosomal cathepsins (CTSB), FN1, and LGALS3BP. These ECM-associated proteins were elevated at the proteome level, but were also differentially expressed in the plaque transcriptome and/or in the secretome of lipid-loaded human vascular SMCs.

Based on our findings, these proteins are promising biomarkers of plaque instability: First, they are locally expressed in the atherosclerotic plaque (transcriptomics/proteomics), with SMCs being a potential source (secretome analysis). Second, they are elevated in the plaques of symptomatic patients and detectable in the circulation, indicating that they may get released from unstable lesions. We provide some evidence that plasma levels of these markers indeed reflect their abundance in plaques; this is supported by previous studies (20–22). Finally, in the prospective community-based Bruneck Study, all identified candidates predicted the development of advanced atherosclerosis in a unique person-based progression model of atherosclerosis. Moreover, strong associations were found with incident cardiovascular events over the 10-year follow-up period, a majority of which derived from plaque rupture and atherothrombosis in both the Bruneck and SAPHIR studies, and a 4-biomarker signature significantly improved risk prediction.

MMP9, a zinc-dependent matrix protease typically produced by macrophages and vascular SMCs, is an established determinant of ECM remodeling in atherosclerosis (23). MMP9 is elevated in advanced atherosclerotic lesions (24) and is positively associated with neutrophil count and morphological characteristics of rupture-prone lesions (25). The protease colocalizes with neutrophils and macrophages at the shoulder region of atherosclerotic plaques and induces collagen breakdown in the fibrous cap, thus contributing to plaque vulnerability and rupture (25, 26). Circulating MMP9 has also been shown to predict clinical events such as stroke (27) and fatal CVD (28). While it is reassuring that the final selection includes established mediators of ECM degeneration like MMP9, omics analyses provide a more integrated assessment of the molecular signature in symptomatic atherosclerosis.

Calprotectin, a heterocomplex of the alarmins S100A8 and S100A9, is mainly produced by myeloid cells such as neutrophils and monocytes and is released in neutrophil extracellular traps (29). Calprotectin promotes migration of phagocytes, triggers endothelial activation, and regulates inflammatory processes by binding to Toll-like receptor 4 and the receptor for advanced glycation end-products. Calprotectin shows increased gene expression in atherosclerotic carotid plaques from patients with recent symptoms (30). In human carotid endarterectomy specimens, levels of calprotectin correlate with features of plaque instability (31). Circulating calprotectin increases early in patients with acute MI and concentrations are highest at the site of coronary occlusion (32). Several studies have shown that calprotectin is associated with the severity of atherosclerosis (33, 34) and the risk of future cardiovascular events (35, 36).

LGALS3BP, also known as MAC-2 binding protein, is a member of the macrophage scavenger receptor cysteine-rich domain superfamily (37). LGALS3BP has been implicated in the regulation of innate immunity (38) and in the modulation of cell-cell and cell-matrix interactions (39). Its role in the vasculature is not well understood, but it has recently been shown to play a proinflammatory role in human venous thrombosis via its binding to galectin 3, enhancing leukocyte adhesion to the vessel wall and increasing IL-6 expression (40). Notably, LGALS3BP is associated with carotid artery disease in patients with human immunodeficiency virus and hepatitis C virus infection (41). An in vitro study revealed the expression of LGALS3BP in proinflammatory M1 macrophages (42). Therefore, LGALS3BP may be a marker of macrophage activation, but LGALS3BP is also produced by vascular SMCs. Its expression by SMCs after lipid loading and the deposition of LGALS3BP in the vascular ECM could affect leukocyte binding and activation in the vessel wall, with implications in atherosclerosis progression as indicated by the association of circulating LGALS3BP with advanced atherosclerosis in the Bruneck Study.

Cathepsins are lysosomal proteases that are thought to play an important role in vascular ECM remodeling and atherogenesis (43). Cathepsins exert both intracellular and extracellular functions. They can degrade ECM components such as collagen and elastin and promote apoptotic processes. Several cathepsins, including cathepsins B and D, have been found to colocalize with macrophages in atherosclerotic lesions (44, 45), and cathepsin G has been found to play an important role in recruiting leukocytes to the arterial walls (46). Interestingly, cathepsin B has recently been identified in a comparison of gene expression between stable and unstable segments of plaque obtained from the same patient (47).

CHI3L1 is a phylogenetically highly conserved protein associated with various inflammatory disorders. The involvement of CHI3L1 in the pathogenesis of atherosclerosis and manifestation of CVD has recently been highlighted. Studies have shown that CHI3L1 levels are associated with both the presence and the extent of coronary artery disease and predict all-cause and cardiovascular mortality (48, 49). In case-control and community-based studies, elevated levels of CHI3L1 were associated with increased risk of stroke, but not MI (17, 50). In carotid endarterectomy specimens, *CHI3L1* mRNA levels are higher in patients with symptomatic than those with asymptomatic disease (51). In contrast to CRP, a nonspecific inflammatory marker produced in the liver, CHI3L1 is locally expressed by macrophages within inflamed arterial tissue (52).

FN1 is a classic marker of wound healing and is produced by activated macrophages, fibroblasts, and SMCs during tissue remodeling. FN1 binds cellular and ECM components (e.g., integrins, TNC, etc.), thereby mediating cell adherence and plasticity. FN1 has been implicated in a number of diseases, including atherosclerosis (53). Interestingly, a recent experimental study showed that FN1 determines the inflammatory responsiveness of endothelial cells to disturbed flow patterns, suggesting an important role of FN1 in the early stage of atherosclerosis (54). Nevertheless, studies investigating the association between circulating FN1 and coronary artery disease have reported conflicting results (53).

Immunohistochemistry of the identified proteins (LGALS3BP, S100A8, S100A9, CHI3L1, MMP9) along with cell markers (CD68: macrophages; CNN and SMA: SMCs) showed localization in the plaque area, linking the plasma biomarkers to the underlying pathology. Additional proteins were identified by one of the three discovery approaches but not confirmed by the others. Interesting examples are extracellular molecules that have been previously implicated in plaque vulnerability, most notably osteopontin (SPP1), which was differentially expressed in our ECM proteomics analysis (FDR <0.01, Figure 1A) and for which a significant correlation in 64 tissue-plasma sample pairs was observed in the Athero-Express cohort (55) (data not shown; *r* = 0.336, *P* = 0.007), providing further evidence that plasma levels are, at least to some extent, indicative of the protein abundance in atherosclerotic plaques. A previous proteomics study of human carotid samples, which unlike our study was not targeted toward the ECM, led to the identification of osteopontin as a prognostic marker for secondary manifestation of atherosclerosis (55).

The present study has notable strengths. First, we applied a proteomics technique optimized to study the ECM and proteins attached to the ECM. The comprehensive analysis of the ECM allows a detailed characterization of the composition and changes of the extracellular environment occurring during atherosclerosis progression and plaque destabilization. Thus far, proteomics studies have used whole tissue lysates (18, 55), and only one study restricted their analysis to proteoglycans of the ECM (19). Second, omics analyses were performed without a priori assumptions, thereby facilitating the potential discovery of biomarker candidates. Third, we combined experimental and epidemiological research. The proteome signature identified was validated in two community-based studies.

A limitation of our study is that omics technologies can result in false positive findings. To overcome this drawback, we considered candidates only if they were detected by ECM proteomics and at least one of the two other discovery approaches. We acknowledge that the list of identified proteins is not exhaustive for all proteins potentially involved in plaque destabilization. Moreover, although there is strong indirect evidence that plasma levels of the identified markers indeed reflect abundance in the plaque, direct support to this concept is still limited. Finally, the Bruneck and SAPHIR studies include only individuals of European descent, and therefore findings can only be generalized to this ethnicity.

In summary, we were able to unravel a proteome signature that is capable to differentiate between atherosclerotic plaques from symptomatic and asymptomatic patients and to predict the development of advanced atherosclerosis and cardiovascular events in the general population. Most proteomics studies to date have searched for biomarkers in plasma. Plasma, however, has the most complex proteome, exceeding the analytical capabilities of current mass spectrometers. Moreover, the origin of biomarkers in plasma can remain uncertain. Instead, we applied a proteomics approach targeted to the ECM of the diseased tissue and of the relevant cell type. One of the remarkable observations in our study is that tissue-based proteomics focusing on extra- rather than intracellular proteins emerged as a highly efficient technique and yielded 30 differentially expressed proteins in a 6-to-6 comparison (after correction for multiple testing). In contrast, comparison of the transcriptome identified only 14 proteins in a much larger collection of 121 carotid plaques. The biomarker candidates were subsequently validated in two prospective community-based studies. In an exploratory plasma proteomics approach utilizing the read-out of the two multiplex assays (CVD and Inflammation Panel, Olink), less than 5% of the 131 proteins were significantly linked to advanced atherogenesis (data not shown), and most of the candidates were involved in coagulation or fibrinolysis (atherothrombosis) or features of plaque instability (e.g., micro-calcification). Thus, our tissue- and cell-based discovery approach resulted in a higher success rate of identifying biomarkers than a plasma proteomics screen of established and putative biomarkers of inflammation and CVD. Further exploration of the function of these candidate proteins in plaque destabilization is merited.

Our findings may well have potential clinical implications. First, the biomarker signature identified improves risk prediction of CVD (relying on plaque rupture/fissuring) and of the progression of advanced atherosclerosis. This concept may be further advanced to aid clinicians with risk stratifications of patients with carotid stenosis. Second, the biomarker signature (i) may be validated as a useful diagnostic tool in the acute management of CVD before markers of tissue damage (e.g., high sensitivity troponins) increase; and (ii) may inform clinicians about an atherothrombotic origin of ischemic stroke.

## Methods

### Carotid endarterectomy samples.

Atherosclerotic plaque samples were collected from 12 patients undergoing carotid endarterectomies (12). Six of those samples were obtained shortly after an acute cerebrovascular event (symptomatic stenosis with ischemic stroke in the ipsilateral carotid territory), and the remaining 6 samples were collected during an elective surgery (asymptomatic stenosis). The patients were age and sex matched (3 females and males in each group; Supplemental Table 1). The samples were immediately snap-frozen and kept in liquid nitrogen for further use.

### Tissue extraction for proteomics.

To remove plasma contaminants, carotid endarterectomy samples were partially thawed and weighed, and approximately 150 mg tissue per sample was placed in ice-cold PBS. The buffer was supplemented with 1% v/v proteinase inhibitor mixture (Sigma-Aldrich). In addition, 25 mM EDTA was added to ensure inhibition of metalloproteinase. While the tissue samples were immersed in the cold saline, they were diced with a scalpel into 8–10 smaller pieces to facilitate the removal of plasma contaminants and the effective extraction of extracellular proteins as described below. The diced samples were first incubated in a 0.5 M NaCl, 10 mM Tris, pH 7.5 buffer supplemented with proteinase and phosphatase inhibitor cocktails as above and 25 mM EDTA. The volume of the buffer was adjusted to 10:1 of the tissue weight (i.e., 100 mg in 1 ml). The samples were mildly vortexed for 4 hours at room temperature (RT). The NaCl extracts were then desalted with centrifugation using desalting columns (Zeba Spin, Pierce Biotechnology). After desalting, the extracts were mixed with 100% acetone (5:1 volume ratio) at –20°C for 16 hours. Proteins were precipitated with centrifugation (16,000 *g* for 45 minutes), and the pellets were vacuum dried and re-dissolved in deglycosylation buffer (see below). Subsequently, the aortic samples were incubated with 0.08% SDS (10:1 buffer volume to tissue weight), including proteinase and phosphatase inhibitor cocktails and 25 mm EDTA. The samples were mildly vortexed, to minimize mechanical disruption of the ECM, for 4 hours at RT. The SDS solution was then removed and stored frozen for later use. Finally, the samples were incubated in a 4 M GuHCl, 50 mM sodium acetate pH 5.8 buffer (5:1 buffer volume to tissue weight), plus proteinase and phosphatase inhibitor cocktails and 25 mM EDTA. pH was adjusted to 5.8 to improve the extractability of proteoglycans (56). The samples were incubated for 48 hours at RT and vortexed vigorously to enhance the mechanical disruption of ECM and ECM-associated proteins. After the denaturing extraction, guanidine was removed by mixing the samples with 100% ethanol (5:1 volume ratio) at –20ºC for 16 hours. Proteins were then precipitated with centrifugation (16,000 *g* for 45 minutes), and the pellets were washed with 90% ethanol, dried, and re-dissolved in deglycosylation buffer. Deglycosylation of the different extracts was achieved in a 150 mM NaCl, 50 mM sodium acetate pH 6.8 buffer supplemented with proteinase inhibitors and 10 mM EDTA for 16 hours at 37°C. The deglycosylation enzymes (0.05 U) were chondroitinase ABC from *Proteus vulgaris*, keratanase from *Bacteroides fragillis*, and heparinase II from *Flavobacterium heparinum*. All enzymes were purchased from Sigma-Aldrich. After deglycosylation, the solutions were centrifuged (16,000 *g* for 10 minutes). Protein concentration was measured by UV absorbance at 280 nm using the Groves formula, [concentration (mg/ml) = (1.55 × A280) – 0.76 × A260], to account for nucleic acid interference (57).

### Proteomics analysis of tissue extracts.

The 0.5 M NaCl and 4 M GuHCl extracts were denatured and reduced in sample buffer containing 100 mM Tris, pH 6.8, 40% glycerol, 0.2% SDS, 2% β-mercaptoethanol, and 0.02% bromophenol blue and boiled at 96°C for 10 minutes (9). 35 μg of protein per sample was loaded and separated on Bis-Tris discontinuous 4%–12% polyacrylamide gradient gels (NuPage, Invitrogen) alongside protein standards (prestained All Blue, Precision Plus, Bio-Rad). Gels were stained using the PlusOne Silver staining Kit (GE Healthcare). Silver staining was used for band staining to avoid cross-contamination with fainter gel bands (58). All gel bands were excised in identical parallel positions across lanes, and no empty gel pieces were left behind. Subsequently, all gel bands were subjected to in-gel tryptic digestion using an Investigator ProGest (Genomic Solutions) robotic digestion system. Tryptic peptides were separated on a nanoflow LC system (ThermoFisher Scientific UltiMate 3000) and eluted with eluent A (2% acetonitrile, 0.1% formic acid in H_2_O) and B (90% acetonitrile, 0.1% formic acid in H_2_O) using a 70-minute gradient (10%–25% B in 35 minutes, 25%–40% B in 5 minutes, 90% B in 10 minutes, and 2% B in 20 minutes). The column (ThermoFisher Scientific PepMap C18, 25-cm length, 75-μm internal diameter, 3-μm particle size) was coupled to a nanospray source (Picoview). During the liquid chromatography–mass spectrometry (LC-MS) run, spectra were collected from a high-mass accuracy analyzer (LTQ Orbitrap XL, ThermoFisher Scientific) using full ion scan mode over the mass-to-charge (*m/z*) range 450–1,600. Tandem MS (MS/MS) was performed on the top 6 ions in each MS scan using the data-dependent acquisition mode with dynamic exclusion enabled. MS/MS peaklists were generated by extract_msn.exe and matched to human database (UniProtKB version 2013_8, 88,378 protein entries; http://www.uniprot.org/) using Mascot (version 2.3.01, Matrix Science). Carboxyamidomethylation of cysteine was chosen as fixed modification, and oxidation of methionine, lysine, and proline was chosen as variable modifications. The variable modifications of lysine and proline were included due to the large quantity of collagens in the samples. The mass tolerance was set at 1.5 AMU for the precursor ions and at 1.0 AMU for fragment ions. Two missed cleavages were allowed. Scaffold (version 4.3.2, Proteome Software Inc.) was used to calculate the normalized spectral counts, and to validate peptide and protein identifications (59, 60). Peptide identifications were accepted if they could be established at greater than 95.0% probability as specified by the Peptide Prophet algorithm (59). Only tryptic peptides were included in the analysis. Protein identifications were accepted if they could be established at greater than 95.0% probability (60) with at least two independent peptides and a mass accuracy of ≤10 ppm of the precursor ion. ECM proteins identified for the first time by proteomics in the vasculature as well as ECM proteins with low spectral counts were further examined to ensure the quality of the identified spectra. The mass spectrometry proteomics data have been deposited to the ProteomeXchange Consortium via the PRIDE partner repository with the dataset identifier PXD005130 and DOI 10.6019/PXD005130.

### Human vascular SMC culture and proteomics analysis.

Human vascular SMCs were obtained from explants of human aortic tissue as previously described (61). SMCs from 6 different donors ranging in age from 20 to 54 years, both male and female, were used. SMCs were cultured in M199 medium (Sigma-Aldrich) supplemented with 20% FBS (Sigma-Aldrich), 2 mM l-glutamine (Gibco), 100 U/ml penicillin, and 100 mg/ml streptomycin, and used between passages 4 and 14. Lipid-loaded SMCs were obtained as previously described (14). The cell layer was lysed using RIPA buffer (Cell Signaling Technology). The serum-free conditioned media were concentrated using an Amicon 3 kDa molecular weight cut-off spin column (Millipore). 30 μg cell layer proteins or 10 μg secreted proteins per sample were denatured and reduced in sample buffer containing 100 mM Tris, pH 6.8, 40% glycerol, 0.2% SDS, 2% β-mercaptoethanol, and 0.02% bromophenol blue and boiled at 96°C for 10 minutes. Samples were loaded, separated, silver stained, digested, and analyzed by LC-MS/MS as described above in *Proteomics analysis of tissue extracts*. The identification was performed with mass tolerance set at 10 ppm for the precursor ions and at 0.8 Da for fragment ions. The mass spectrometry proteomics data have been deposited to the ProteomeXchange Consortium via the PRIDE partner repository with the dataset identifier PXD005130 and DOI 10.6019/PXD005130.

### Western blots.

For immunoblotting, we followed standard protocols (58). The 0.5 M NaCl and SDS extracts of the 12 endarterectomy samples were aliquoted and mixed with denaturing sample buffer and boiled at 96°C for 10 minutes. On separate 15-well 4%–12% polyacrylamide gels (NuPAGE, Invitrogen) for each extract, 30 μg protein was loaded and separated alongside protein standards. The separated proteins were then transferred onto a nitrocellulose membrane. Following transfer, the membranes were blocked with 5% fat-free milk powder in PBS and probed overnight at 4°C with primary antibodies in 5% bovine serum albumin at a 1:1,500 dilution.

The primary antibodies used for 0.5 M NaCl extracts were FN1 (SC-56391, clone SPM246, Santa Cruz Biotechnology Inc.), SPP1 (SC-20788, rabbit polyclonal, Santa Cruz Biotechnology Inc.), and fibromodulin (FMOD) (SC-25857, goat polyclonal, Santa Cruz Biotechnology Inc.). Primary antibodies for integrin beta 1 (ITB1) (SC-73610, clone 102DF5, Santa Cruz Biotechnology Inc.), CD68 (SC-70761, clone 3F103, Santa Cruz Biotechnology Inc.), CD31 (SC-31045, goat polyclonal, Santa Cruz Biotechnology Inc.), and SMA (A5691, clone 1A4, Sigma-Aldrich) were used on the SDS extracts. For human SMC experiments, the blots were also probed with antibodies to MMP9 (ab38898, rabbit polyclonal, Abcam), tenascin C (ab6393, clone BC-24, Abcam), fibronectin (SC-56391, clone SPM246, Santa Cruz Biotechnology Inc.), and LGALS3BP (70R-6085, rabbit polyclonal, Fitzgerald).

The membranes were then treated in appropriate horseradish peroxidase–conjugated secondary antibodies at a 1:2,000 dilution. Enhanced ChemiLuminescence (ECL, GE Healthcare) was used for imaging, and the exposed films were developed on a Xograph processor. ImageJ software (V.1.4.3.67; NIH) was used for densitometry of each lane of the developed films.

### MMP9 zymography.

MMP9 activity of the 0.5 M NaCl extracts of the 12 carotid endarterectomy specimens was determined by SDS-PAGE gelatin zymography. The extracts were mixed with non-reducing sample buffer and electrophoresed in SDS-PAGE gels containing 0.1% gelatin. Gels were incubated in the presence of metalloproteinase activating buffer (50 mM Tris, pH 7.4, and 200 mM NaCl with 2.5% Triton X-100) at RT for 1 hour and subsequently in metalloproteinase activating buffer at 37°C overnight. The gels were stained with Coomassie blue. Proteolysis was detected as a clear band against a blue background. The activity of MMP9 was determined by densitometry. A similar approach was used for conditioned media of SMCs. MMP2 was used as loading control.

### MMP9 digestion.

Human aortic explants were incubated with 50 pM MMP9 (Calbiochem) or left untreated, for 24 hours at 37°C. The degradation of FN1, LGALS3BP, and TNC was examined by immunoblotting as described above in *Western blots*.

### Gene expression in carotid endarterectomies.

Patients undergoing surgery for symptomatic or asymptomatic, high-grade (>50% NASCET) (62) carotid stenosis at the Department of Vascular Surgery, Karolinska University Hospital, were consecutively enrolled as part of the BiKE study and clinical data recorded on admission. Symptoms of plaque instability were defined as transitory ischemic attack, minor stroke, and amaurosis fugax. Patients without qualifying symptoms within 6 months prior to surgery were categorized as asymptomatic, and indication for carotid endarterectomy was based on results from the Asymptomatic Carotid Surgery Trial (63). Plaque classification was based solely on patient’s symptoms. The BiKE study cohort demographics, details of sample processing, and full microarray and statistical analyses have been previously described (11, 64), and the microarray dataset is available from the NCBI’s Gene Expression Omnibus (GSE21545). Briefly, global gene expression profiles have been analyzed by Affymetrix HG-U133 plus 2.0 Genechip microarrays in 127 patients’ plaque tissues (*n* = 87 symptomatic and *n* = 40 asymptomatic) and *n* = 10 non-atherosclerotic (normal) arteries.

### Immunohistochemistry.

For the carotid plaques, all immunohistochemistry reagents were from Biocare Medical. Tissues were fixed for 48 hours in 4% Zn formaldehyde at RT and paraffin-embedded. Isotype rabbit and mouse IgG were used as negative controls. In brief, 5-μm sections were deparaffinized in Tissue Clear (Histolab) and rehydrated in graded ethanol. For antigen retrieval, slides were subjected to high-pressure boiling in DIVA buffer (pH 6.0). After blocking with Background Sniper, primary antibodies were diluted in Da Vinci Green solution, applied on slides and incubated at RT for 1 hour. For colocalizations, antibodies for cell-specific markers were used: anti-SMA (M0851, clone 1A4, Dako), CNN1 (calponin, ab110128, goat polyclonal, Abcam), CD68 (M0876, clone PG-M1, Dako). Other antibodies against candidate biomarker proteins used in the study were: anti-LGALS3BP (70R-6085, rabbit polyclonal, R&D Systems), CHI3L1 (human chitinase 3-like-1) (AF2599, goat polyclonal, R&D Systems), S100A8 (ab92331, clone RPR3554 rabbit, Abcam), S100A9 (ab92507, clone EPR3555 rabbit, Abcam), and MMP9 (ab38898, rabbit polyclonal, Abcam). A double-stain probe-polymer system containing alkaline phosphatase and horseradish peroxidase was applied, with subsequent detection using Warp Red and Vina Green. Slides were counterstained with Hematoxylin QS (Vector Laboratories), dehydrated, and mounted in Pertex (Histolab). Images were taken using an automated ScanScope slidescanner. Scale bars are included in the images as indicated in the Figure legends.

In human aortic explants, after rehydration, the 3-μm sections were unmasked using hot sodium citrate buffer. Sections were washed 3 times with PBS-Tween (PBST) and incubated with primary antibodies of LGALS3BP (70R-6085, Fitzgerald) or species-matched isotopes overnight at 4°C after blocking with 10% FBS in PBST for 1 hour. Following 3 washes in PBST, 5 minutes each, sections were incubated for 1 hour at RT with the secondary antibody (Alexa Fluor 594, Life Technologies) in 10% FBS/PBST, according to the source of the primary antibody. Nuclei were visualized using DAPI (1:1,000 dilution) for 10 minutes. Then sections were mounted on VectaMount (Vector Laboratories). Sections were visualized with a 20× CFI S Plan Fluor ELWD ADM objective using an inverted Nikon NI-E microscope equipped with a Yokogawa CSU-X1 spinning disk confocal unit and an Andor iXon 3 EM-CCD camera. Images were acquired using NIS-elements 4.0 software.

### Study populations for epidemiological validation.

The Bruneck study is a prospective community-based survey on the epidemiology and pathogenesis of atherosclerosis that started in 1990 (16, 65–69). The study population was recruited as a sex- and age-stratified random sample of all residents aged 40 to 79 living in Bruneck (*n* = 4,739), Northern Italy. The present analysis focuses on the second quinquennial follow-up examination, with full data available in 685 individuals and a 10-year follow-up period (years 2000–2010).

All risk factors were assessed by means of validated standard procedures described previously (65–69). In brief, body mass index was calculated as weight divided by height squared (kg/m^2^). Hypertension was defined as blood pressure ≥140/90 mmHg (mean of 3 independent readings obtained with a standard mercury sphygmomanometer after at least 10 minutes of rest) or the use of antihypertensive drugs. Lifetime smoking was assessed as pack-years. Diabetes was defined based on ADA criteria (70). Blood samples were drawn in 2000 after an overnight fast and 12 hours of abstinence from smoking, and laboratory parameters were measured by standard assays.

The composite CVD end point was composed of ischemic stroke, MI, and vascular death. Ischemic stroke was classified according to the criteria of the National Survey of Stroke (71). MI was deemed confirmed when World Health Organization criteria for definite disease status were met. Vascular mortality included deaths from ischemic stroke, MI, and rupture of aortic aneurysms and sudden cardiac deaths (72). Ascertainment of events did not rely on hospital discharge codes or the patient’s self-report but on a review of medical records provided by the general practitioners, death certificates, and Bruneck Hospital files, as well as the extensive clinical and laboratory examinations performed as part of the study protocols. Major advantages of the Bruneck Study are that virtually all subjects living in the Bruneck area were referred to the local hospital and that the network existing between the local hospital and the general practitioners allowed retrieval of practically all medical information on persons living in the area.

At each study visit, participants underwent bilateral carotid duplex sonography using a 10-MHz transducer and a 5-MHz Doppler (16, 62, 64). All subjects were examined in supine position. The scanning protocol involved 4 segments of the right and left carotid artery: proximal common carotid artery (15–30 mm proximal to the carotid bulb), distal common carotid artery (<15 mm proximal to the carotid bulb), proximal internal carotid artery (carotid bulb and initial 10 mm of the vessel), distal internal carotid artery (>10 mm above the flow divider) (Supplemental Figure 9). A plaque was defined as a focal structure encroaching into the arterial lumen with a widening of the vessel wall of at least 0.5 mm relative to surrounding segments. The extent of carotid atherosclerosis was quantified by a scoring system adding the maximum axial thickness of atherosclerotic plaques (in mm) on the near and far wall at each of the 8 vessel segments.

A person-based atherosclerosis progression model (16, 67) was developed and validated in the Bruneck study that allowed differentiation between early and advanced stages in atherosclerosis development: (a) Early atherogenesis subsumes the manifestation of new plaques and/or nonstenotic progression of existing plaques. Main characteristics are a slow and continuous plaque growth usually affecting more than one plaque simultaneously and accompanied by a compensatory or even overcompensatory enlargement of the vessel at the plaque site. The term “incipient atherosclerosis” was used for the development of first plaques in subjects free of atherosclerosis at baseline. (b) Advanced complicated atherogenesis was assumed when the relative increase in the maximum plaque diameter exceeded twice the measurement error for the method (internal carotid artery, 24.8%; common carotid artery, 17%) (16) and a lumen narrowing of at least 40% was achieved. This process is characterized by a usually solitary prominent increase in plaque size and the absence of vascular remodeling, resulting in significant lumen compromise (Supplemental Figure 10). From a mechanistic perspective, it refers to plaque destabilization and subsequent atherothrombosis (16). The two progression categories were highly reproducible (κ coefficients >0.8 [*n* = 100]). Risk profiles differ significantly between the two stages of carotid artery disease, with early atherosclerosis relying on standard risk factors and advanced atherosclerosis being mainly related to markers reflecting plaque vulnerability or enhanced prothrombotic activity (69).

The SAPHIR study is a prospective cohort study conducted in 1,770 healthy unrelated subjects (663 females and 1,107 males aged 39–67 years) who were recruited by health screening programs in large companies in and around the city of Salzburg, Austria (73). The cohort was first examined in the years 1999–2002, reexamined in 2003–2008, and followed until September 2013. At baseline and during the follow-up visit, all participants were subjected to a comprehensive examination, including a detailed medical history and physical, instrumental, and laboratory investigations (73, 74). Definitions of clinical variables were the same as in the Bruneck study.

For the current analysis, we used a case-cohort design. Of the 1,770 participants in the SAPHIR study, we first excluded 433 participants because they had no plasma sample, had an acute infection at enrollment (defined as CRP >4 mg/dl), had a history of CVD at enrollment, or were lost to follow-up. Among the remaining 1,337 participants, we selected all participants with incident primary CVD event (defined as MI, ischemic stroke, or vascular death), all participants with CVD other than stroke and MI, plus an age- and sex-matched subcohort of 151 participants. In total, there were 58 incident events of the primary CVD outcome (6 in the subcohort) and 82 incident cases of additional CVD outcomes (15 in the subcohort).

### Plasma samples from patients with symptomatic carotid stenosis.

Plasma samples were drawn from 17 consecutive patients with symptomatic carotid stenosis. The mean age was 70.8 ± 9.0 years, and 70.6% were male. Plasma levels of MMP9 and CALP were measured using ELISA (MMP9 from R&D Systems, DMP900; and S100A8/A9 from BMA Biomedicals).

### Biomarker measurements in plasma.

In the Bruneck year 2000 evaluation and the SAPHIR study, the following proteins were measured by ELISA in plasma samples according to the manufacturers’ instructions: MMP9 from R&D Systems (DMP900); CHI3L1 from R&D Systems (DC3L10); LGALS3BP from R&D Systems (DGBP30); S100A8/A9 from BMA Biomedicals. Additional plasma proteins were analyzed using proximity extension assays (CVD I and Inflammation I panels, Olink) as previously published (15).

### Statistics.

Proteomic differential expression was assessed using the normalized spectral abundance factor–power law global error model (NSAF-PLGEM) (75). NSAF was calculated for each protein detected. Spectral count values of 0 were replaced by an empirically derived fractional value. The value was calculated by detaining the smallest value between 0 and 1 that provided the best fit to a normal distribution. The fit to a normal distribution was determined with a Shapiro-Wilk test. For proteomics of human vascular SMCs, the differential expression in the 9 proteins in common was assessed by a 2-tailed, paired *t* test with a Bonferroni correction, significance <5% family-wise error rate. The disease association enrichment was performed using WebGestalt with all identified proteins as background (76).

From the identified plaque ECM proteins, gene expression was available for 120 corresponding genes (a subset of 231 probes) from the BiKE study described in Perisic et al. (11), and only those were used for transcriptomic differential expression analysis. The linear models for microarray data (limma) method was used to detect differential expression at the probe level, and a FDR threshold of 10% was applied (77). All correlation values, including the coexpression networks, are Pearson correlations with associated *P* values. For the coexpression networks, the associated *P* values were corrected for using *Q* values, and a 20% FDR threshold was applied. The protein correlations were calculated after applying the NSAF normalization.

In the Bruneck study, associations of baseline ECM protein levels with early and advanced stages of atherosclerosis progression were analyzed by means of unconditional logistic regression analysis. The main analyses were adjusted for age and sex. Multivariable models additionally controlled for standard vascular risk factors, baseline atherosclerosis, and high-sensitivity CRP level. This model should be viewed as conservative because it considered adjustment for systemic inflammation and atherosclerosis burden, and some of the proinflammatory risk factors may well impact plaque stability by our candidate proteins. A final signature of biomarkers was identified by a forward-stepwise selection procedure applying standard inclusion and exclusion criteria. Cox proportional hazard models with adjustment for age and sex or multivariable adjustment (plus vascular risk factors and high-sensitivity CRP level) were used to assess whether baseline ECM protein levels were associated with new-onset CVD (2000 to 2010). We detected no departure from the proportional hazards assumption by inspecting Schoenfeld residuals and checking the parallelity of log-log survival plots. We analyzed the case-cohort data from the SAPHIR study using Cox proportional hazard models with Prentice weights and robust standard errors (78).

This study used distinct approaches to assess the incremental predictive value afforded by markers of plaque vulnerability. First, the improvement in risk discrimination resulting from adding markers of plaque vulnerability to a model containing the standard Framingham risk factors was quantified using the C-statistic (logistic regression analysis on advanced atherosclerosis) and Harrell’s C-index (Cox models on incident CVD). The C-index is suitable for time-to-event data and gives the probability that the model correctly predicts the order of failure of randomly selected pairs of individuals. A C-index/C-statistic of 1.0 indicates perfect prediction of the order of failure, whereas a value of 0.5 is achieved purely by chance. The 95% CIs for C-indices and their changes were derived from jackknife standard errors. Comparison of the C-statistic/C-index for models including and not including vulnerability biomarkers (individual components and the 4-biomarker signature) was performed according to the method of DeLong (79) and with the Stata procedure somersd. Second, we evaluated whether the 4-biomarker signature helps correctly classify participants into categories of predicted CVD risk. Using the 10-year risk categories <5%, 5% to <7.5%, and ≥7.5%, the categorical net reclassification improvement (NRI) (80) was calculated. Separate analyses focus on the continuous NRI, which does not depend on the choice of categories, but deems any change in predicted risk in the correct direction as appropriate. Finally, we calculated the integrated discrimination improvement (IDI), which integrates the NRI over all possible cut-offs of predicted risk and mathematically corresponds to the difference in discrimination slopes of the 2 models in comparison (81, 82). Statistical analyses were performed with SPSS 22.0 and Stata 12.0 MP software packages. All reported *P* values are 2-sided.

### Study approval.

All clinical investigations were conducted according to Declaration of Helsinki principles. All participants gave written informed consent prior to inclusion in the study. The study of the carotid endarterectomy samples was approved by the local Research Ethics Committee (London, United Kingdom, Research Ethics Committee, REC reference number 08/H0706/129). The BiKE study was approved by the Ethical Committee of Northern Stockholm, and all samples were collected with informed consent from patients, organ donors, or their guardians. Aortic samples were collected from patients undergoing aortic surgery at St. George’s Hospital, and the Regional Ethics Committee Board approved all procedures involving use of human aortic tissues (London, United Kingdom, Research Ethics Committee, REC reference number 08/H0803/257). The human vascular SMCs were obtained as previously described, with the appropriate approval by the local research ethics committee (60). The Bruneck study was approved by the Ethics Committees of Verona and Bolzano (Italy). The SAPHIR study was approved by the Ethical Committee of Salzburg (Austria). The study using plasma samples from symptomatic patients was approved by the Ethics Committee of the Medical University of Innsbruck (Austria).

## Author contributions

SRL was involved with the study design; analyzed and integrated the proteomic, transcriptomic, and SMC experiments; and wrote portions of and edited the manuscript. KW performed the Bruneck cohort phenotyping; analyzed the epidemiological data; and wrote portions on the manuscript. AD was involved with the study design; designed and performed the plaque proteomics experiments; and wrote portions of the manuscript. LPM and UH provided the transcriptomic and respective clinical data. PS performed the epidemiological validation of the proteomics experiments. JBB performed the immunoblot validations and provided ECM expertise. ML, RL, and CM performed immunohistochemistry and immunofluorescence analysis. AK and GR provided lipid-loaded human vascular SMCs, and samples from the Bruneck cohort. XY performed plaque proteomics experiments and provided proteomics expertise. TB and GS performed ELISA measurements. LK, BI, and BP provided access to samples from the SAPHIR cohort and made critical revisions to the manuscript. PW performed the statistical analysis for risk prediction. JS, AHD, and CM obtained carotid endarterectomies and characterized the plaque data. GP provided data from the Athero-Express cohort. CMS provided expertise and advice on human vascular SMCs. JW designed and ran the Bruneck cohort. SK performed the epidemiological analysis. MM was involved with the study design, secured funding, and wrote the majority of the manuscript.

## Supplementary Material

Supplemental data

ICMJE disclosure forms

## Figures and Tables

**Table 2:**
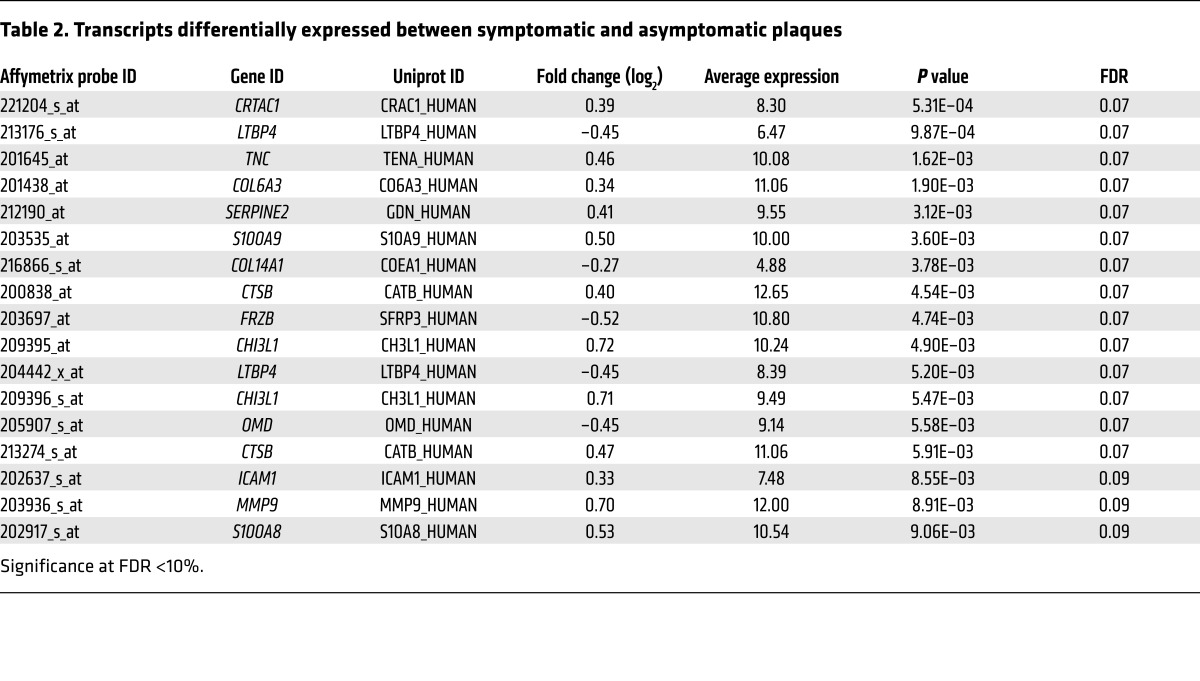
Transcripts differentially expressed between symptomatic and asymptomatic plaques

**Table 1:**
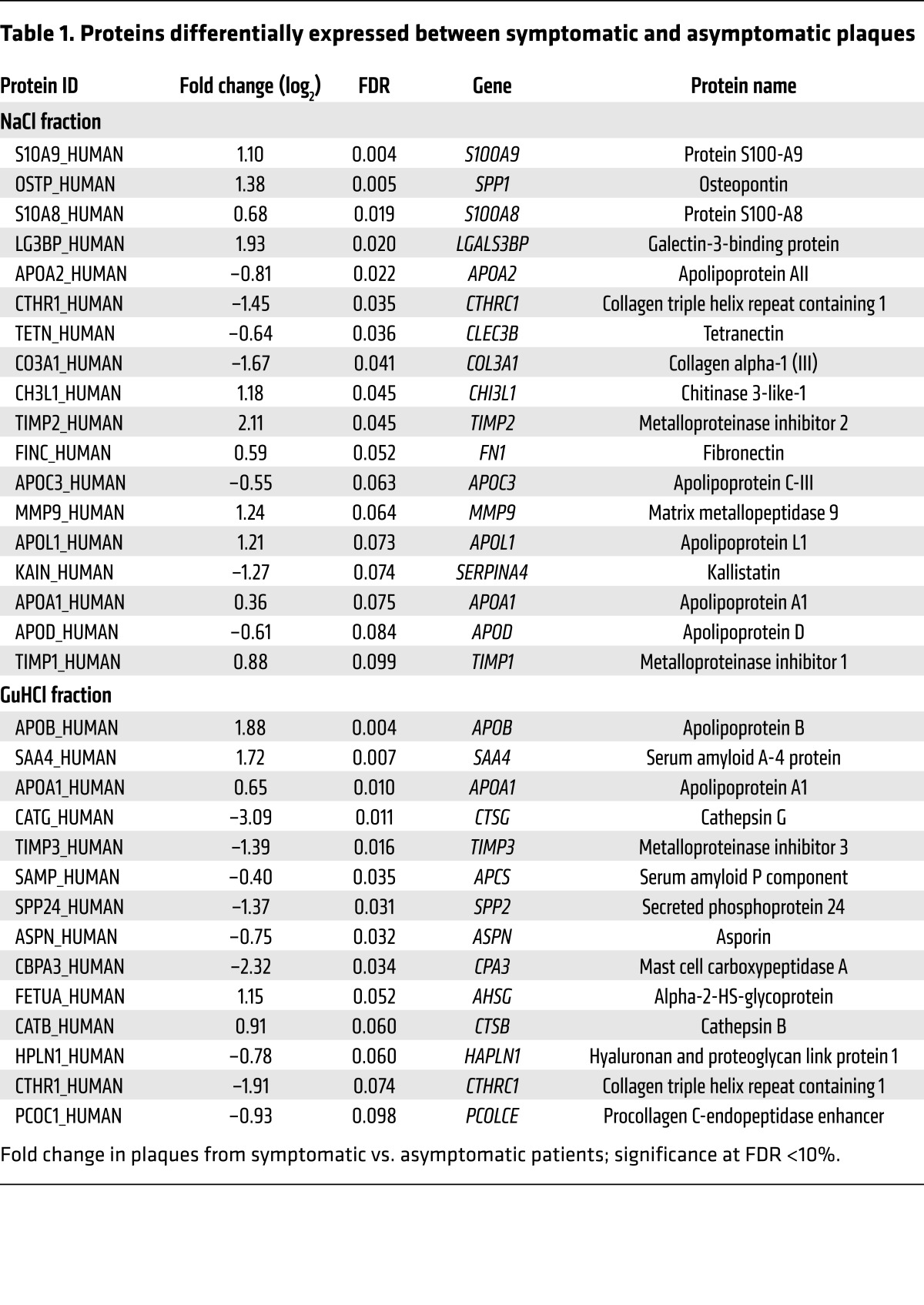
Proteins differentially expressed between symptomatic and asymptomatic plaques
