# Supplementary material for: Extracellular matrix proteomics identifies molecular signature of symptomatic carotid plaques
Source: J Clin Invest. 2017 Mar 20;127(4):1546–60. doi: 10.1172/JCI86924 (PMC5373893; doi:10.1172/JCI86924)

ONLINE SUPPLEMENT

Extracellular Matrix Proteomics Identifies Molecular Signature of Symptomatic Carotid Plaques

Sarah R. Langley[*1,2], Karin Willeit[*3], Athanasios Didangelos[*1], Ljubica Perisic Matic[4], Philipp Skroblin[1], Javier Barallobre-Barreiro[1], Mariette Lengquist[4], Gregor Rungger[5], Alexander Kapustin[1], Ludmilla Kedenko[6], Chris Molenaar[7], Ruifang Lu[1], Temo Barwari[1], Gonca Suna[1], Xiaoke Yin[1], Bernhard Iglseder[8], Bernhard Paulweber[6], Peter Willeit[3,9], Joseph Shalhoub[10], Gerard Pasterkamp[11], Alun H. Davies[10], Claudia Monaco[12], Ulf Hedin[4], Catherine M. Shanahan[1], Johann Willeit[3], Stefan Kiechl[3], Manuel Mayr[1]

From the [1]King's British Heart Foundation Centre, King's College London, London, United Kingdom; [2]Duke-NUS Medical School, Singapore, Republic of Singapore; [3]Department of Neurology, Medical University Innsbruck, Austria; [4]Department of Molecular Medicine and Surgery, Vascular Surgery, Karolinksa University, Stockholm, Sweden; [5]Department of Neurology, Bruneck Hospital, Bruneck, Italy; [6]First Department of Internal Medicine, Paracelsus Medical University, Salzburg, Austria; [7]Nikon Imaging Centre, King's College London, London, United Kingdom; [8]Department of Geriatric Medicine, Paracelsus Medical University, Salzburg, Austria; [9]Department of Public Health and Primary Care, University of Cambridge, Cambridge, United Kingdom; [10]Department of Surgery & Cancer, Imperial College London, London, United Kingdom; [11]University Medical Center Utrecht, Utrecht, The Netherlands; [12]Kennedy Institute, University of Oxford, Oxford, United Kingdom

*** Equal contribution**

Supplementary Table 1. Clinical characteristics of the patient cohort undergoing carotid endarterectomy.

	**Asymptomatic (n=6)**	**Symptomatic (n=6)**
Gender (M:F)	3:3	3:3
Age	70±6.1	75±5.3
Symptomatic (%)	0%	100%
Type of Event	n/a	Stroke*
Side of Body (Left:Right)	n/a	4:2
Diabetes (%)	2 (33.3%)	1 (16.7%)
Hypertension (%)	6 (100%)	6 (100%)
PAD (%)	2 (33.3%)	3 (50%)
Smoker (%)	2 (33.3%)	5 (83.3%)
Aspirin (%)	6 (100%)	6 (100%)
Clopidogrel (%)	1 (16.7%)	2 (33.3%)
Dipyridamole (%)	0 (0%)	1 (16.7%)
Statin (%)	6 (100%)	5 (83.3%)

There were no significant differences using the Student's t test or Chi-squared test.
* ischemic stroke in the ipsilateral carotid territory

Supplementary Table 2. List of ECM proteins identified after proteomics analysis of carotid endarterectomies from symptomatic and asymptomatic patients.

			Guanidine extracts				NaCl extracts				Transcriptomics		
Protein name	Uniprot ID	Gene ID	Symptomatic	Asymptomatic	Log2 (Fold Change)	FDR	Symptomatic	Asymptomatic	Log2 (Fold Change)	FDR	Affymetrix Probe ID	Log2 (Fold Change)	FDR
Adipocyte enhancer-binding protein 1	AEBP1_HUMAN	AEBP1	196.3±32.9	203.8±41	-0.05	0.808	89.2±11.9	90.5±23.1	-0.02	0.910	205040_at	-0.05	0.782
Adiponectin	ADIPO_HUMAN	ADIPOQ	-	-	-	-	5.5±2.1	6.2±2.6	-0.17	0.602	-	-	-
Aggrecan	PGCA_HUMAN	ACAN	66±60.1	94.8±61.8	-0.52	0.249	2.8±4.9	2±1.9	0.50	0.538	-	-	-
Agrin	AGRIN_HUMAN	AGRN	2.2±4.4	2.2±1.5	0.00	0.970	-	-	-	-	212285_s_at; 217419_x_at	-0.11	0.305
Alpha-1-acid glycoprotein 1	A1AG1_HUMAN	ORM1	-	-	-	-	41.8±11	36±5.4	0.22	0.270	205040_at	0.03	0.752
Alpha-1-acid glycoprotein 2	A1AG2_HUMAN	ORM2	-	-	-	-	13.5±7.2	11.7±1.9	0.21	0.405	214465_at	-0.04	0.662
Alpha-1-B glycoprotein	A1BG_HUMAN	A1BG	15.2±4.4	14.2±3.8	0.10	0.757	-	-	-	-	229819_at	0.10	0.295
Alpha-2-HS-glycoprotein	FETUA_HUMAN	AHSG	17.3±20.5	7.8±11.7	1.15	**0.052**	-	-	-	-	-	-	-
Angiotensinogen	ANGT_HUMAN	AGT	-	-	-	-	6.7±4.3	8.8±1.7	-0.41	0.325	202834_at	-0.06	0.774
Annexin A2	ANXA2_HUMAN	ANXA2	-	-	-	-	79±7	87.8±18.4	-0.15	0.381	-	-	-
Apolipoprotein A	APOA_HUMAN	LPA	7.7±5.4	13.5±13.3	-0.82	0.410	4.8±5.5	9.7±7.8	-1.00	0.258	209978_s_at	0.00	0.995
Apolipoprotein A-I	APOA1_HUMAN	APOA1	273.7±202.4	174.8±98.9	0.65	**0.011**	123.8±38.6	96.3±14.5	0.36	**0.075**	217073_x_at; 204450_x_at	-0.04	0.661
Apolipoprotein A-II	APOA2_HUMAN	APOA2	5±4	3.5±1.4	0.51	0.200	10.2±2.8	17.8±10.1	-0.81	**0.022**	219465_at; 219466_s_at	0.06	0.483
Apolipoprotein A-IV	APOA4_HUMAN	APOA4	207.3±103.3	176.7±47.3	0.23	0.199	36.2±11.9	49±7.6	-0.44	0.121	206894_at	0.04	0.765
Apolipoprotein B	APOB_HUMAN	APOB	500.3±780.5	136±216.6	1.88	**0.004**	248.2±101.1	226.2±74.3	0.13	0.579	205108_s_at	-0.16	0.388
Apolipoprotein C-I (truncated)	K7ERI9_HUMAN	APOC1	6.2±7.6	4.3±2.9	0.51	0.161	-	-	-	-	-	-	-
Apolipoprotein C-II	APOC2_HUMAN	APOC2	-	-	-	-	6.5±5.3	8.3±5.1	-0.36	0.206	204561_x_at	0.27	0.324
Apolipoprotein C-III	APOC3_HUMAN	APOC3	10±3.7	14.7±5.2	-0.55	0.122	11.5±2.7	16.8±11.9	-0.55	**0.063**	205820_s_at	-0.03	0.817
Apolipoprotein D	APOD_HUMAN	APOD	17.7±10.8	21.5±8.6	-0.28	0.302	10.2±6.2	15.5±3.2	-0.61	**0.084**	201525_at	-0.07	0.614
Apolipoprotein E	APOE_HUMAN	APOE	252.8±89.6	282±79.7	-0.16	0.264	65.3±16	83.5±30.9	-0.35	0.115	203381_s_at; 203382_s_at	0.46	0.129
Apolipoprotein H	APOH_HUMAN	APOH	48.8±29.6	56.3±30.9	-0.21	0.407	36.2±12	44.2±9.1	-0.29	0.231	205216_s_at	0.01	0.878
Apolipoprotein L, 1	APOL1_HUMAN	APOL1	-	-	-	-	5±1.9	2.2±1.2	1.21	**0.073**	-	-	-
Asporin	ASPN_HUMAN	ASPN	57.7±26.6	96.7±35	-0.75	**0.032**	-	-	-	-	224396_s_at	-0.52	0.129
Biglycan	PGS1_HUMAN	BGN	361.5±76.7	405.2±43.1	-0.16	0.232	18.8±8.6	14.5±10.9	0.38	0.231	201262_s_at	-0.41	0.129
Carboxypeptidase B2	CBPB2_HUMAN	CPB2	6±7.8	6.8±6.3	-0.19	0.705	-	-	-	-	-	-	-
Cartilage acidic protein 1	CRAC1_HUMAN	CRTAC1	1.7±1.2	2.8±3.3	-0.77	0.387	-	-	-	-	221204_s_at; 1555958_at	0.39	**0.074**
Cartilage oligomeric matrix protein	COMP_HUMAN	COMP	1.5±2.5	3.3±7.2	-1.15	0.243	3.7±5.6	4.8±6.6	-0.40	0.450	205713_s_at	0.38	0.145
Cathepsin B	CATB_HUMAN	CTSB	22.2±19.6	11.8±14.3	0.91	**0.060**	28.5±13.5	24±13.5	0.25	0.317	200838_at; 213274_s_at; 200839_s_at; 213275_x_at; 227961_at	0.40	**0.074**
Cathepsin D	CATD_HUMAN	CTSD	17.5±14.6	15.7±9.5	0.16	0.613	26.8±9.2	19.8±7.1	0.44	0.170	200766_at	0.30	0.141
Cathepsin F	CATF_HUMAN	CTSF	2.2±2.3	3.3±2.4	-0.62	0.333	-	-	-	-	-	-	-
Cathepsin G	CATG_HUMAN	CTSG	0.7±0.8	5.7±4.4	-3.09	**0.011**	-	-	-	-	205653_at	0.25	0.261
Cathepsin S	CATS_HUMAN	CTSS	-	-	-	-	11.3±6.6	6.8±8.1	0.73	**0.100**	202902_s_at	0.39	0.129
Cell surface glycoprotein MUC18	MUC18_HUMAN	MCAM	-	-	-	-	0.7±0.8	1.7±2	-1.32	0.164	209086_x_at; 210869_s_at	-0.26	0.225
Chitinase-3-like protein 1	CH3L1_HUMAN	CHI3L1	-	-	-	-	2.2±3.5	0.5±0.8	1.18	**0.045**	209395_at; 209396_s_at	0.72	**0.074**
Clusterin	CLUS_HUMAN	CLU	258.3±48.1	289.2±53.7	-0.16	0.325	-	-	-	-	222043_at; 208791_at; 208792_s_at	-0.26	0.187
Collagen alpha-1 (I)	CO1A1_HUMAN	COL1A1	602.7±179.1	687±196.8	-0.19	0.275	27.5±6.8	31.3±10.1	-0.19	0.576	202311_s_at; 202312_s_at	0.42	0.129
Collagen alpha-1 (III)	CO3A1_HUMAN	COL3A1	84.2±35.2	99.2±47.4	-0.24	0.466	5.5±6.7	17.5±8.3	-1.67	**0.041**	-	-	-
Collagen alpha-1 (IV)	CO4A1_HUMAN	COL4A1	24±7.7	24.2±10.3	-0.01	0.984	-	-	-	-	211981_at	-0.03	0.882
Collagen alpha-1 (V)	CO5A1_HUMAN	COL5A1	65.2±26.7	72.8±20.8	-0.16	0.663	-	-	-	-	212488_at; 203325_s_at; 212489_at	0.09	0.515
Collagen alpha-1 (VI)	CO6A1_HUMAN	COL6A1	386.8±44.1	412.3±64.4	-0.09	0.557	5±4.1	5.2±4.8	-0.05	0.913	212937_s_at; 212938_at; 212940_at; 212091_s_at	-0.32	0.129
Collagen alpha-1 (VIII)	CO8A1_HUMAN	COL8A1	2±3.3	2.5±1.4	-0.32	0.666	-	-	-	-	214587_at	-0.41	0.156
Collagen alpha-1 (XIV)	COEA1_HUMAN	COL14A1	194.7±124.6	229±54.5	-0.23	0.355	173.5±39.9	198.7±24	-0.20	0.386	216865_at; 216866_s_at	-0.27	**0.074**
Collagen alpha-1 (XV)	COFA1_HUMAN	COL15A1	46±18.3	47.3±14	-0.04	0.918	12.7±4.8	8.3±4.4	0.60	0.258	-	-	-
Collagen alpha-1 (XVIII)	COIA1_HUMAN	COL18A1	120.5±44.3	175.3±30.2	-0.54	0.145	42.2±16.2	54.5±10.7	-0.37	0.298	209081_s_at; 209082_s_at	-0.28	0.163
Collagen alpha-1(XII)	COCA1_HUMAN	COL12A1	131.7±67.4	159.8±70.5	-0.28	0.436	206.7±54	189.8±35.7	0.12	0.586	231879_at; 231766_s_at; 234951_s_at	-0.21	0.269
Collagen alpha-2 (I)	CO1A2_HUMAN	COL1A2	504.2±127.9	578±175.7	-0.20	0.306	24.7±4.7	23.5±3.6	0.07	0.814	-	-	-
Collagen alpha-2 (IV)	CO4A2_HUMAN	COL4A2	38.3±13.5	54.2±24.3	-0.50	0.257	-	-	-	-	211966_at	-0.36	0.129
Collagen alpha-2 (V)	CO5A2_HUMAN	COL5A2	79.5±39.6	78.8±51.1	0.01	0.930	-	-	-	-	221729_at; 221730_at	0.14	0.414
Collagen alpha-2 (VI)	CO6A2_HUMAN	COL6A2	145±49.1	177.3±41.6	-0.29	0.244	3.8±2.5	4.7±3.1	-0.28	0.598	209156_s_at	-0.18	0.188
Collagen alpha-2 (VIII)	CO8A2_HUMAN	COL8A2	2.5±3.7	3.3±3.1	-0.41	0.587	-	-	-	-	52651_at; 221900_at	-0.17	0.156
Collagen alpha-3 (VI)	CO6A3_HUMAN	COL6A3	1144.5±120.5	1174.8±152.3	-0.04	0.828	36.3±12.4	38±19.4	-0.06	0.855	201438_at	0.34	**0.074**
Collagen triple helix repeat containing 1	CTHR1_HUMAN	CTHRC1	0.7±1	2.5±1.6	-1.91	**0.074**	1.8±2.6	5±2.5	-1.45	**0.035**	-	-	-
Decorin	PGS2_HUMAN	DCN	87.5±14.5	111±47	-0.34	0.144	2.5±2.3	3.2±3.7	-0.34	0.468	209335_at	0.23	0.261
Dermatopontin	DERM_HUMAN	DPT	70.8±25.4	88.7±13.9	-0.32	0.129	5.7±2.9	6.7±4.8	-0.23	0.455	213068_at; 213071_at; 207977_s_at	-0.33	0.129
EMILIN-2	EMIL2_HUMAN	EMILIN2	2.2±3.9	0.7±0.8	1.70	0.200	-	-	-	-	224374_s_at; 242288_s_at; 221980_at	0.39	0.128
Extracellular superoxide dismutase [Cu-Zn]	SODE_HUMAN	SOD3	29±10.7	36.3±6.2	-0.33	0.224	38.8±12.1	38.8±11.2	0.00	0.961	205236_x_at	-0.30	0.129
Fibrillin 1	FBN1_HUMAN	FBN1	4.3±5.9	0.8±1.3	2.38	0.161	-	-	-	-	202765_s_at	0.01	0.987
Fibromodulin	FMOD_HUMAN	FMOD	25±12.2	21.7±8.9	0.21	0.481	5.8±10.2	6±5.5	-0.04	0.898	202709_at	-0.10	0.656
Fibronectin	FINC_HUMAN	FN1	956±178.5	906.2±97.5	0.08	0.613	386.7±139.9	256±74.5	0.59	**0.052**	214702_at	0.10	0.584
Fibulin 1	FBLN1_HUMAN	FBLN1	13±16.1	14.5±6.3	-0.16	0.733	43.5±14.5	45.2±7.1	-0.05	0.805	207835_at; 201787_at; 202995_s_at	-0.09	0.422
Fibulin 2	FBLN2_HUMAN	FBLN2	-	-	-	-	31±16.5	34.2±16.8	-0.14	0.640	203886_s_at	0.12	0.470
Fibulin 3	FBLN3_HUMAN	EFEMP1	-	-	-	-	189.5±35.1	200.5±21.6	-0.08	0.554	201843_s_at	-0.07	0.661
Fibulin 5	FBLN5_HUMAN	FBLN5	4±4.4	3.5±3	0.19	0.703	21.5±7.6	24.3±7.8	-0.18	0.497	203088_at	-0.40	0.142
Galectin 1	LEG1_HUMAN	LGALS1	21.3±7.4	25.2±8.4	-0.24	0.336	47±7.6	46±9.4	0.03	0.799	-	-	-
Galectin 3	LEG3_HUMAN	LGALS3	40.3±10.3	38.3±10.9	0.07	0.703	10±2.9	8.8±5.5	0.18	0.513	-	-	-
Galectin-3-binding protein	LG3BP_HUMAN	LGALS3BP	45.5±17.4	33.5±21.2	0.44	0.200	9.5±11.4	2.5±2.8	1.93	**0.020**	200923_at	-0.17	0.206
Glia-derived nexin	GDN_HUMAN	SERPINE2	9.7±9.2	10.5±3.8	-0.12	0.775	-	-	-	-	212190_at	0.41	**0.074**
Hemicentin 1	HMCN1_HUMAN	HMCN1	-	-	-	-	7.8±8.1	6±2.3	0.38	0.604	235944_at	-0.42	0.129

Hyaluronan and proteoglycan link protein 1	HPLN1_HUMAN	HAPLN1	23.7±17.9	40.5±22.3	-0.78	**0.060**	-	-	-	-	230895_at; 230204_at; 205523_at; 205524_s_at	-0.27	0.237
Hyaluronan and proteoglycan link protein 3	HPLN3_HUMAN	HAPLN3	26.2±17.5	31.7±13.1	-0.28	0.397	-	-	-	-	-	-	-
Inactive carboxypeptidase-like protein X2	CPXM2_HUMAN	CPXM2	13.2±5.2	14.2±5.5	-0.11	0.789	-	-	-	-	236144_at	-0.32	0.129
Insulin-like growth factor binding protein 7	IBP7_HUMAN	IGFBP7	48.7±15.7	44.7±11.8	0.12	0.548	26±11.6	29±1.1	-0.16	0.471	-	-	-
Insulin-like growth factor-binding protein complex acid labile subunit	ALS_HUMAN	IGFALS	-	-	-	-	8.2±2.2	7.5±2.1	0.12	0.719	215712_s_at	-0.08	0.580
Intercellular adhesion molecule 1	ICAM1_HUMAN	ICAM1	3±2.7	1±1.7	1.58	0.126	-	-	-	-	202637_s_at; 202638_s_at	0.33	**0.093**
Kallistatin	KAIN_HUMAN	SERPINA4	3±3.5	5.2±8.5	-0.78	0.250	2±1.9	4.8±1.9	-1.27	**0.074**	213874_at	-0.10	0.397
Lactadherin	MFGM_HUMAN	MFGE8	132±12.6	154.7±37.2	-0.23	0.255	-	-	-	-	-	-	-
Laminin subunit alpha-5	LAMA5_HUMAN	LAMA5	10.5±11.3	16.5±6.3	-0.65	0.437	-	-	-	-	210150_s_at	-0.18	0.216
Laminin subunit beta-1	LAMB1_HUMAN	LAMB1	10±8.9	5.2±3	0.95	0.253	-	-	-	-	-	-	-
Laminin subunit beta-2	LAMB2_HUMAN	LAMB2	32±7.2	44.8±16.2	-0.49	0.327	-	-	-	-	216264_s_at	-0.07	0.618
Laminin subunit gamma-1	LAMC1_HUMAN	LAMC1	53.5±19.3	57.2±15.3	-0.10	0.801	-	-	-	-	-	-	-
Latent-transforming growth factor beta-binding protein 1	LTBP1_HUMAN	LTBP1	12.5±7.5	14.7±5	-0.23	0.684	-	-	-	-	-	-	-
Latent-transforming growth factor beta-binding protein 2	LTBP2_HUMAN	LTBP2	36.3±31.7	36.8±11.1	-0.02	0.967	47.2±10	50.8±7.8	-0.11	0.712	223690_at; 223690_at	-0.29	0.129
Latent-transforming growth factor beta-binding protein 4	LTBP4_HUMAN	LTBP4	17.2±7.6	16.2±2.9	0.09	0.832	5.5±7.6	5.7±2.9	-0.04	0.935	204442_x_at; 213176_s_at	-0.45	**0.074**
Lumican	LUM_HUMAN	LUM	206.2±54.8	241±33.9	-0.23	0.179	48.2±9.4	49.3±11.8	-0.03	0.837	229554_at	0.34	0.145
Macrophage migration inhibitory factor (glycosylation-inhibiting factor)	MIF_HUMAN	MIF	-	-	-	-	17.5±9	15.5±2.6	0.18	0.375	-	-	-
Mast cell carboxypeptidase A	CBPA3_HUMAN	CPA3	1.2±2.4	5.8±5.9	-2.32	**0.034**	-	-	-	-	205624_at	0.29	0.372
Matrilin-2	MATN2_HUMAN	MATN2	1.7±1.6	1.8±2.9	-0.14	0.892	-	-	-	-	-	-	-
Matrix Gla protein	MGP_HUMAN	MGP	14.8±7.6	16.3±9.2	-0.14	0.555	23.2±5.8	24.2±8.8	-0.06	0.694	-	-	-
Matrix metallopeptidase 12	MMP12_HUMAN	MMP12	-	-	-	-	5±5.1	5.8±6.1	-0.22	0.576	-	-	-
Matrix metallopeptidase 2	MMP2_HUMAN	MMP2	-	-	-	-	5.3±3.6	5.5±2.1	-0.04	0.907	-	-	-
Matrix metallopeptidase 9	MMP9_HUMAN	MMP9	3±3.2	1.5±2.3	1.00	0.264	9.8±5.7	4.2±4.8	1.24	**0.064**	203936_s_at	0.70	**0.093**
Metalloproteinase inhibitor 1	TIMP1_HUMAN	TIMP1	12.2±8.9	10.2±8.8	0.26	0.415	4±2.8	2.2±1.3	0.88	**0.099**	-	-	-
Metalloproteinase inhibitor 2	TIMP2_HUMAN	TIMP2	2.7±1.8	3.5±1.6	-0.39	0.460	5.7±3.3	2.5±1.9	2.11	**0.045**	203167_at	0.19	0.181
Metalloproteinase inhibitor 3	TIMP3_HUMAN	TIMP3	7.3±5.5	19.2±11.8	-1.39	**0.016**	-	-	-	-	201147_s_at; 201148_s_at; 201149_s_at	0.33	0.145
Mimecan	MIME_HUMAN	OGN	140.7±30.1	170.5±20.3	-0.28	0.145	8±2.8	8.3±3.2	-0.06	0.833	218730_s_at	-0.46	0.129
Neutrophil defensin 1	DEF1_HUMAN	DEFA1	7.8±3.6	10.3±6.3	-0.40	0.194	-	-	-	-	205033_s_at	0.09	0.782
Nidogen 1	NID1_HUMAN	NID1	19.8±13.1	34.7±11.6	-0.81	0.142	-	-	-	-	202007_at; 202008_s_at	-0.10	0.641
Nidogen 2	NID2_HUMAN	NID2	8.5±4.7	10±4.4	-0.23	0.681	-	-	-	-	204114_at	0.27	0.241
Osteomodulin	OMD_HUMAN	OMD	4.5±6.2	4.7±5.6	-0.05	0.906	-	-	-	-	205907_s_at; 205908_s_at	-0.45	**0.074**
Osteopontin	OSTP_HUMAN	SPP1	12±13.3	6.8±5.7	0.81	0.114	41.7±27.1	16±8.9	1.38	**0.005**	-	-	-
Periostin	POSTN_HUMAN	POSTN	170.3±82.2	141±71.4	0.27	0.249	19.5±5.6	18.3±7.3	0.09	0.752	1555778_a_at	0.11	0.618
Perlecan	PGBM_HUMAN	HSPG2	355.8±138.9	477.8±157.4	-0.43	0.180	104.5±34.2	81.3±12	0.36	0.323	201654_s_at	-0.12	0.397
Plasma protease C1 inhibitor	IC1_HuMAN	SERPING1	24.2±21	26±8	-0.11	0.760	-	-	-	-	-	-	-
Podocan	PODN_HUMAN	PODN	11.7±4.4	16.8±4.8	-0.53	0.254	1±1.7	0.3±0.5	1.58	0.159	226522_at	-0.17	0.142
Procollagen C-endopeptidase enhancer	PCOC1_HUMAN	PCOLCE	10±3.3	19±6.4	-0.93	**0.098**	47.8±12.8	44.7±12	0.10	0.610	-	-	-
Prolargin	PRELP_HUMAN	PRELP	347.5±79.5	404.2±50.5	-0.22	0.154	-	-	-	-	37022_at	-0.18	0.145
Prosaposin	SAP_HUMAN	PSAP	-	-	-	-	15±4.5	20.5±7.3	-0.45	0.208	200866_s_at	0.25	0.145
Protein APOC4-APOC2	K7ER74_HUMAN	APOC4-APOC2	1.2±1.6	1.3±2.4	-0.19	0.764	-	-	-	-	-	-	-
Protein S100-A10	S10AA_HUMAN	S100A10	0.5±0.8	0.7±0.8	-0.41	0.545	6.3±5	7.7±6.6	-0.28	0.298	-	-	-
Protein S100-A11	S10AB_HUMAN	S100A11	-	-	-	-	13±2	12.3±4.5	0.08	0.673	-	-	-
Protein S100-A4	S10A4_HUMAN	S100A4	1.3±1	1.3±1.6	0.00	0.992	12.2±5.6	10.5±3.3	0.21	0.333	-	-	-
Protein S100-A6	S10A6_HUMAN	S100A6	-	-	-	-	5.7±4.5	6±5.2	-0.08	0.712	-	-	-
Protein S100-A8	S10A8_HUMAN	S100A8	-	-	-	-	29.2±13.8	18.2±7.1	0.68	**0.019**	202917_s_at	0.53	**0.093**
Protein S100-A9	S10A9_HUMAN	S100A9	5.3±6.9	5.3±7.6	0.00	0.975	36.2±24.1	16.8±18.6	1.10	**0.004**	203535_at	0.50	**0.074**
Secreted frizzled-related protein 3	SFRP3_HUMAN	FRZB	4±3	5±2.4	-0.32	0.502	-	-	-	-	244419_at; 203698_s_at	-0.52	**0.074**
Secreted phosphoprotein 24	SPP24_HUMAN	SPP2	4.3±4.2	11.2±13.4	-1.37	**0.036**	-	-	-	-	214478_at	0.00	0.979
Serine protease HTRA1	HTRA1_HUMAN	HTRA1	72±22.7	93.3±8.1	-0.37	0.182	-	-	-	-	-	-	-
Serum amyloid A-4 protein	SAA4_HUMAN	SAA2-SAA4	12.7±13.1	3.8±5.6	1.72	**0.008**	-	-	-	-	207096_at	0.06	0.439
Serum amyloid P component	SAMP_HUMAN	APCS	284.8±73.9	374.7±64.4	-0.40	**0.035**	96.5±24.8	111.5±25	-0.21	0.199	206350_at	0.00	0.998
Spondin 1	SPON1_HUMAN	SPON1	-	-	-	-	1.8±3.6	3.2±4.6	-0.79	0.269	-	-	-
Stromal cell-derived factor 1	SDF1_HUMAN	CXCL12	4.2±1.9	3.8±0.8	0.12	0.686	-	-	-	-	203666_at	-0.03	0.859
Target of Nesh-SH3	TARSH_HUMAN	ABI3BP	-	-	-	-	4.2±4	5±6.1	-0.26	0.621	223395_at	0.14	0.439
Tenascin C	TENA_HUMAN	TNC	501.2±161.5	398.2±109	0.33	0.179	91.2±33.9	62.3±31.7	0.55	0.146	201645_at	0.46	**0.074**
Tenascin XB	TENX_HUMAN	TNXB	108.3±64.1	121±58.1	-0.16	0.662	-	-	-	-	217413_s_at	0.17	0.175
Tetranectin	TETN_HUMAN	CLEC3B	16±8.5	21±7	-0.39	0.208	26.7±10.2	41.7±9.3	-0.64	**0.036**	205200_at	0.14	0.430
Thrombospondin 1	TSP1_HUMAN	THBS1	47±26.2	40.5±32.6	0.21	0.524	202±98.2	173.8±99.9	0.22	0.289	201108_s_at; 201109_s_at; 235086_at	0.35	0.145
thrombospondin 2	TSP2_HUMAN	THBS2	68.7±53.9	45.8±26.4	0.58	0.154	87.7±30.6	64.3±23.9	0.45	0.150	-	-	-
Thrombospondin 3	TSP3_HUMAN	THBS3	-	-	-	-	0.7±1.2	2.2±2	-1.70	0.123	-	-	-
Thrombospondin 4	TSP4_HUMAN	THBS4	-	-	-	-	2±2.2	2.2±2.7	-0.12	0.845	204776_at	-0.07	0.414
Transforming growth factor-beta-induced protein ig-h3	BGH3_HUMAN	TGFBI	36±18.7	39.3±11.1	-0.13	0.695	-	-	-	-	-	-	-
Transthyretin	TTHY_HUMAN	TTR	27.3±16.7	22.7±13.5	0.27	0.264	-	-	-	-	209660_at	-0.01	0.962
Tryptase beta-2	TRYB2_HUMAN	TPSB2	6±5.6	6.3±3.4	-0.08	0.857	-	-	-	-	-	-	-
Tubulointerstitial nephritis antigen-like 1	TINAL_HUMAN	TINAGL1	59.3±15.4	66.5±4	-0.16	0.473	-	-	-	-	219058_x_at	-0.20	0.129
Versican	CSPG2_HUMAN	VCAN	471.8±111.6	535.7±79.4	-0.18	0.421	74±34.5	74±13.8	0.00	0.960	211571_s_at	-0.22	0.175
Vitronectin	VTNC_HUMAN	VTN	222.3±44.6	238.7±49.8	-0.10	0.528	65.5±33	48±19.8	0.45	0.108	204534_at	0.04	0.596
von Willebrand factor	VWF_HUMAN	VWF	-	-	-	-	2.8±4.1	4.2±3.9	-0.56	0.495	202112_at	0.30	0.162

Supplementary Table 3. List of ECM proteins identified in human vascular SMC layers.

Identified ECM proteins	Accession Number	Entry name	MW (kD)	Identified in plaque
72 kDa type IV collagenase	P08253	MMP2_HUMAN	74	yes
Adipocyte plasma membrane-associated protein	Q9HDC9	APMAP_HUMAN	46	
Agrin	O00468	AGRIN_HUMAN	217	yes
Alpha-2-HS-glycoprotein	P02765	FETUA_HUMAN	39	
Alpha-2-macroglobulin receptor-associated protein	P30533	AMRP_HUMAN	41	
Annexin A2	P07355	ANXA2_HUMAN	39	yes
Basement membrane-specific heparan sulfate proteoglycan core protein	P98160	PGBM_HUMAN	469	yes
Biglycan	P21810	PGS1_HUMAN	42	yes
Cadherin-13	P55290	CAD13_HUMAN	78	
Cadherin-2	P19022	CADH2_HUMAN	100	
Calpain small subunit 1	P04632	CPNS1_HUMAN	28	
Calumenin	O43852	CALU_HUMAN	37	
Carboxypeptidase Q	Q9Y646	CBPQ_HUMAN	52	
Cartilage-associated protein	O75718	CRTAP_HUMAN	47	
Cathepsin B	P07858	CATB_HUMAN	38	yes
Cathepsin D	P07339	CATD_HUMAN	45	yes
Cathepsin L1	P07711	CATL1_HUMAN	38	
Cathepsin Z	Q9UBR2	CATZ_HUMAN	34	
Cell migration-inducing and hyaluronan-binding protein	Q8WUJ3	CEMIP_HUMAN	153	
Collagen alpha-1(I)	P02452	CO1A1_HUMAN	139	yes
Collagen alpha-1(III)	P02461	CO3A1_HUMAN	139	yes
Collagen alpha-1(V)	P20908	CO5A1_HUMAN	184	yes
Collagen alpha-1(VI)	P12109	CO6A1_HUMAN	109	yes
Collagen alpha-2(I)	P08123	CO1A2_HUMAN	129	yes
Collagen alpha-2(V)	P05997	CO5A2_HUMAN	145	
Collagen alpha-2(VI)	P12110	CO6A2_HUMAN	109	yes
Collagen alpha-3(VI)	P12111	CO6A3_HUMAN	344	yes
Connective tissue growth factor	P29279	CTGF_HUMAN	38	
EMILIN-1	Q9Y6C2	EMIL1_HUMAN	107	
Epididymal secretory protein E1	P61916	NPC2_HUMAN	17	
Fibrillin-1	P35555	FBN1_HUMAN	312	yes
Fibroblast growth factor 2	P09038	FGF2_HUMAN	31	
Fibroleukin	Q14314	FGL2_HUMAN	50	
Fibronectin	P02751	FINC_HUMAN	263	yes
Fibulin-1	P23142	FBLN1_HUMAN	77	yes
Fibulin-2	P98095	FBLN2_HUMAN	127	
Follistatin-related protein 1	Q12841	FSTL1_HUMAN	35	
Galectin-1	P09382	LEG1_HUMAN	15	yes
Galectin-3	P17931	LEG3_HUMAN	26	yes
Galectin-3-binding protein	Q08380	LG3BP_HUMAN	65	yes
Gelsolin	P06396	GELS_HUMAN	86	
Glypican-1	P35052	GPC1_HUMAN	62	
Growth/differentiation factor 15	Q99988	GDF15_HUMAN	34	
Hepatoma-derived growth factor	P51858	HDGF_HUMAN	27	
Insulin-like growth factor-binding protein 5	P24593	IBP5_HUMAN	31	
Insulin-like growth factor-binding protein 7	Q16270	IBP7_HUMAN	29	yes
Intercellular adhesion molecule 1	P05362	ICAM1_HUMAN	58	yes
Interstitial collagenase	P03956	MMP1_HUMAN	54	
Isoform 2 of Glia-derived nexin	P07093	GDN_HUMAN	44	yes
Lactadherin	Q08431	MFGM_HUMAN	43	yes
Leukocyte elastase inhibitor	P30740	ILEU_HUMAN	43	
Macrophage migration inhibitory factor	P14174	MIF_HUMAN	12	yes
Mammalian ependymin-related protein 1	Q9UM22	EPDR1_HUMAN	25	
Matrix Gla protein	P08493	MGP_HUMAN	12	
Metalloproteinase inhibitor 1	P01033	TIMP1_HUMAN	23	yes
Metalloproteinase inhibitor 2	P16035	TIMP2_HUMAN	24	yes
Metalloproteinase inhibitor 3	P35625	TIMP3_HUMAN	24	
Neudesin	Q9UMX5	NENF_HUMAN	19	
Nucleobindin-1	Q02818	NUCB1_HUMAN	54	
Nucleobindin-2	P80303	NUCB2_HUMAN	50	
Pentraxin-related protein PTX3	P26022	PTX3_HUMAN	42	
Perilipin-2	Q99541	PLIN2_HUMAN	48	
Periostin	Q15063	POSTN_HUMAN	93	
Plasminogen activator inhibitor 1	P05121	PAI1_HUMAN	45	
Procollagen C-endopeptidase enhancer 1	Q15113	PCOC1_HUMAN	48	yes
Procollagen galactosyltransferase 1	Q8NBJ5	GT251_HUMAN	72	
Procollagen-lysine,2-oxoglutarate 5-dioxygenase 1	Q02809	PLOD1_HUMAN	84	
Procollagen-lysine,2-oxoglutarate 5-dioxygenase 2	O00469	PLOD2_HUMAN	85	
Procollagen-lysine,2-oxoglutarate 5-dioxygenase 3	O60568	PLOD3_HUMAN	85	
Prolow-density lipoprotein receptor-related protein 1	Q07954	LRP1_HUMAN	505	
Prosaposin	P07602	SAP_HUMAN	58	
Protein S100-A10	P60903	S10AA_HUMAN	11	yes
Protein S100-A11	P31949	S10AB_HUMAN	12	yes
Protein S100-A6	P06703	S10A6_HUMAN	10	yes
Protein S100-A7	P31151	S10A7_HUMAN	11	
Protein S100-A8	P05109	S10A8_HUMAN	11	yes
Puromycin-sensitive aminopeptidase	P55786	PSA_HUMAN	103	
Serine protease HTRA1	Q92743	HTRA1_HUMAN	51	yes
Serpin B6	P35237	SPB6_HUMAN	43	
Serpin H1	P50454	SERPH_HUMAN	46	
SPARC	P09486	SPRC_HUMAN	35	
Tenascin	P24821	TENA_HUMAN	241	yes
Testican-1	Q08629	TICN1_HUMAN	49	
Thrombospondin-1	P07996	TSP1_HUMAN	129	yes
Thrombospondin-2	P35442	TSP2_HUMAN	130	yes
Thymosin beta-4	P62328	TYB4_HUMAN	5	
Transforming growth factor-beta-induced protein ig-h3	Q15582	BGH3_HUMAN	75	yes
UPF0556 protein C19orf10	Q969H8	CS010_HUMAN	19	
Vasorin	Q6EMK4	VASN_HUMAN	72	
Versican core protein	P13611	CSPG2_HUMAN	373	yes
Vesicle-associated membrane protein 3	Q15836	VAMP3_HUMAN	11	
Vesicle-associated membrane protein-associated protein A	Q9P0L0	VAPA_HUMAN	28	
Vinculin	P18206	VINC_HUMAN	124	

Supplementary Table 4. ECM proteins identified in the secretomes of control and lipid-loaded human vascular SMCs.

Identified ECM Proteins (94)	Accession Number	Uniprot Name	MW (kD)	Control Average± SD (n=4)	Lipid Loaded Average± SD (n=4)	Ratio (log2)	t-test p-value	Identified in plaque
72 kDa type IV collagenase	P08253	MMP2_HUMAN	74	40.4±20.0	47.5±14.2	0.23	0.529	yes
Adipocyte enhancer-binding protein 1	Q8IUX7	AEBP1_HUMAN	131	0.6±0.7	5.5±4.1	3.17	0.133	yes
Agrin	O00468	AGRIN_HUMAN	215	3.8±2.8	8.1±3.3	1.09	0.240	yes
Alpha-1-acid glycoprotein 1	P02763	A1AG1_HUMAN	24	2.9±5.7	0.0±0.0	n/a	0.391	
Alpha-1-acid glycoprotein 2	P19652	A1AG2_HUMAN	24	1.5±2.9	0.0±0.0	n/a	0.391	
Aminopeptidase N	P15144	AMPN_HUMAN	110	0.2±0.3	1.0±0.9	2.65	0.123	
Amyloid beta A4 protein	P05067	A4_HUMAN	87	0.5±1.1	4.0±0.4	2.89	0.008	
Annexin A2	P07355	ANXA2_HUMAN	39	37.7±6.4	49.6±11.9	0.40	0.272	yes
Apolipoprotein D	P05090	APOD_HUMAN	21	3.1±3.2	0.0±0.0	n/a	0.147	
Apolipoprotein E	P02649	APOE_HUMAN	36	0.5±1.1	1.3±2.1	1.27	0.614	
Biglycan	P21810	PGS1_HUMAN	42	9.3±4.9	16.3±9.9	0.81	0.127	yes
Cadherin-13	P55290	CAD13_HUMAN	78	0.6±0.7	3.3±2.7	2.55	0.079	
Cadherin-2	P19022	CADH2_HUMAN	100	0.0±0.0	0.5±0.7	n/a	0.244	
Calmodulin	P62158	CALM_HUMAN	17	6.2±4.8	10.3±4.3	0.74	0.112	
Calnexin	P27824	CALX_HUMAN	68	0.0±0.0	0.5±1.0	n/a	0.391	
**Cathepsin B ***	**P07858**	**CATB_HUMAN**	**38**	**4.9±3.5**	**8.1±3.7**	**0.73**	**0.084**	**yes**
Cathepsin D	P07339	CATD_HUMAN	45	1.4±2.9	6.5±3.8	2.18	0.133	yes
Clusterin	P10909	CLUS_HUMAN	52	6.2±7.0	8.5±7.7	0.47	0.226	yes
Collagen alpha-1(I)	P02452	CO1A1_HUMAN	139	216.2±93.7	289.6±91.3	0.42	0.006	yes
**Collagen alpha-1(III) ***	**P02461**	**CO3A1_HUMAN**	**139**	**41.4±20.1**	**28.9±10.8**	**-0.52**	**0.144**	**yes**
Collagen alpha-1(V)	P20908	CO5A1_HUMAN	184	0.0±0.0	2.7±2.1	n/a	0.082	yes
Collagen alpha-1(VI)	P12109	CO6A1_HUMAN	109	22.9±14.2	26.5±19.1	0.21	0.472	yes
Collagen alpha-1(XII)	Q99715	COCA1_HUMAN	333	20.7±10.5	35.1±28.3	0.76	0.229	yes
Collagen alpha-1(XV)	P39059	COFA1_HUMAN	142	0.0±0.0	1.2±2.4	n/a	0.391	
Collagen alpha-2(I)	P08123	CO1A2_HUMAN	129	374.1±177.1	330.8±112.1	-0.18	0.449	yes
Collagen alpha-2(IV)	P08572	CO4A2_HUMAN	168	8.5±3.2	7.6±5.3	-0.16	0.484	yes
Collagen alpha-2(VI)	P12110	CO6A2_HUMAN	109	3.9±3.9	3.7±2.2	-0.09	0.844	yes
Collagen alpha-3(VI)	P12111	CO6A3_HUMAN	344	12.3±16.5	24.5±17.4	0.99	0.007	yes
Collagen alpha-5(IV)	P29400	CO4A5_HUMAN	161	1.2±1.4	2.6±2.8	1.11	0.227	
Connective tissue growth factor	P29279	CTGF_HUMAN	38	4.4±4.1	9.5±10.4	1.10	0.497	
Decorin	P07585	PGS2_HUMAN	40	11.6±14	10.1±10.6	-0.21	0.686	yes
Endoplasmin	P14625	ENPL_HUMAN	92	8.5±2.9	14.1±4.5	0.74	0.114	
Epididymal secretory protein E1	P61916	NPC2_HUMAN	17	1.6±1.9	3.3±2.4	1.05	0.105	
Extracellular matrix protein 1	Q16610	ECM1_HUMAN	61	1.2±2.4	0.4±0.5	-1.65	0.479	
Extracellular superoxide dismutase [Cu-Zn]	P08294	SODE_HUMAN	26	8.2±11.4	12.0±13.2	0.54	0.061	yes
Fibrillin-1	P35555	FBN1_HUMAN	312	24.3±11.4	28.9±8.1	0.25	0.657	yes
**Fibronectin ***	**P02751**	**FINC_HUMAN**	**263**	**314.7±36.5**	**301.9±60.5**	**-0.06**	**0.368**	**yes**
Fibulin-1	P23142	FBLN1_HUMAN	77	3.9±4.0	10.2±12.8	1.38	0.258	yes
Fibulin-2	P98095	FBLN2_HUMAN	127	5.6±7.4	2.9±5.3	-0.93	0.184	
Fibulin-3	Q12805	FBLN3_HUMAN	55	26.4±13.7	39.6±13.1	0.59	0.179	yes
Fibulin-4	O95967	FBLN4_HUMAN	49	1.8±2.2	0.9±1.1	-1.06	0.193	
Follistatin	P19883	FST_HUMAN	38	6.3±5.4	3.2±3.7	-0.98	0.324	
Follistatin-related protein 1	Q12841	FSTL1_HUMAN	35	22.0±6.9	21.4±9.7	-0.04	0.902	
Galectin-3	P17931	LEG3_HUMAN	26	0.0±0.0	2.1±2.5	n/a	0.182	yes
**Galectin-3-binding protein ***	**Q08380**	**LG3BP_HUMAN**	**65**	**4.4±2.2**	**8.8±1.7**	**0.99**	**0.005**	**yes**
Gelsolin	P06396	GELS_HUMAN	86	1.5±1.2	2.0±2.6	0.44	0.597	
Glypican-1	P35052	GPC1_HUMAN	62	0.6±0.7	2.6±1.4	2.22	0.059	
Glypican-6	Q9Y625	GPC6_HUMAN	63	0.0±0.0	1.4±1.8	n/a	0.224	
Hemopexin	P02790	HEMO_HUMAN	52	0.9±1.9	0.0±0.0	n/a	0.391	
Hyaluronan and proteoglycan link protein 1	P10915	HPLN1_HUMAN	40	0.9±1.8	4.4±8.7	2.27	0.391	
Insulin-like growth factor-binding protein 4	P22692	IBP4_HUMAN	28	9.9±12.5	6.5±4.9	-0.60	0.698	
Insulin-like growth factor-binding protein 5	P24593	IBP5_HUMAN	31	3.5±4.0	2.9±2.6	-0.27	0.746	
Insulin-like growth factor-binding protein 6	P24592	IBP6_HUMAN	25	6.6±8.5	3.9±2.7	-0.77	0.448	
Insulin-like growth factor-binding protein 7	Q16270	IBP7_HUMAN	29	49.4±19.1	33.6±14.8	-0.55	0.382	yes
Integrin alpha-2	P17301	ITA2_HUMAN	129	2.7±3.2	5.1±3.9	0.91	0.122	
Isoform 2 of Glia-derived nexin	P07093	GDN_HUMAN	44	32.5±24.9	66.7±31.9	1.04	0.570	yes
Interstitial collagenase	P03956	MMP1_HUMAN	54	24.7±28.3	28.7±35	0.22	0.637	
Lactadherin	Q08431	MFGM_HUMAN	43	4.7±5.6	15.2±23.9	1.68	0.361	yes
Laminin subunit alpha-4	Q16363	LAMA4_HUMAN	203	4.9±6.2	5.5±5.7	0.17	0.818	
Laminin subunit beta-1	P07942	LAMB1_HUMAN	198	3.3±2.9	3.8±2.9	0.22	0.634	yes
Laminin subunit gamma-1	P11047	LAMC1_HUMAN	178	3.8±4.4	5.3±3.7	0.50	0.326	yes
Latent-transforming growth factor beta-binding protein 1	Q14766	LTBP1_HUMAN	187	3.3±3.9	4.0±3.2	0.29	0.638	
Latent-transforming growth factor beta-binding protein 2	Q14767	LTBP2_HUMAN	195	9.4±2.6	13.4±2.0	0.52	0.081	yes
Lumican	P51884	LUM_HUMAN	38	4.2±3.7	5.3±4.2	0.34	0.544	yes
Lysyl oxidase homolog 2	Q9Y4K0	LOXL2_HUMAN	87	5.9±5.3	7.3±1.5	0.31	0.560	
**Metalloproteinase inhibitor 1 ***	**P01033**	**TIMP1_HUMAN**	**23**	**25.3±14.5**	**25.5±6.4**	**0.01**	**0.978**	**yes**
**Metalloproteinase inhibitor 2 ***	**P16035**	**TIMP2_HUMAN**	**24**	**23.7±6.8**	**18.1±3.9**	**-0.38**	**0.289**	**yes**
Pappalysin-1	Q13219	PAPP1_HUMAN	181	2.9±3.4	2.2±2.8	-0.43	0.444	
Pentraxin-related protein PTX3	P26022	PTX3_HUMAN	42	24.9±22.1	51.5±31.6	1.05	0.027	
Periostin	B1ALD8	B1ALD8_HUMAN	90	56.9±23.3	42.7±22.9	-0.41	0.318	
Perlecan	P98160	PGBM_HUMAN	469	58.0±13.4	60.3±28.7	0.05	0.790	yes
Plasminogen activator inhibitor 1	P05121	PAI1_HUMAN	45	32.1±30.3	61±54.2	0.93	0.107	
Proactivator polypeptide	P07602	SAP_HUMAN	58	0.0±0.0	7.8±6.7	n/a	0.103	
**Procollagen C-endopeptidase enhancer 1 ***	**Q15113**	**PCOC1_HUMAN**	**48**	**20.6±9.3**	**19.4±5.8**	**-0.09**	**0.799**	**yes**
Procollagen-lysine,2-oxoglutarate 5-dioxygenase 1	Q02809	PLOD1_HUMAN	84	3.8±4.0	6.3±4.2	0.74	0.202	
Procollagen-lysine,2-oxoglutarate 5-dioxygenase 3	O60568	PLOD3_HUMAN	85	0.0±0.0	0.3±0.7	n/a	0.391	
Prolow-density lipoprotein receptor-related protein 1	Q07954	LRP1_HUMAN	505	0.0±0.0	2.1±3.6	n/a	0.320	
Proteasome subunit beta type-5	P28074	PSB5_HUMAN	28	3.2±3.8	2.9±1.9	-0.14	0.853	
Protein S100-A7	P31151	S10A7_HUMAN	11	4.4±4.3	1.4±1.2	-1.71	0.237	
**Protein S100-A8 ***	**P05109**	**S10A8_HUMAN**	**11**	**6.3±8.0**	**1.7±1.5**	**-1.87**	**0.285**	**yes**
**Protein S100-A9 ***	**P06702**	**S10A9_HUMAN**	**13**	**14.8±7.5**	**9.2±10.1**	**-0.69**	**0.300**	**yes**
Protein-lysine 6-oxidase	P28300	LYOX_HUMAN	47	10.9±9.0	5.5±3.2	-0.98	0.296	
Retinol-binding protein 4	P02753	RET4_HUMAN	23	1.0±1.9	0.0±0.0	n/a	0.391	
Serine protease HTRA1	Q92743	HTRA1_HUMAN	51	1.2±1.4	2.5±3.4	1.05	0.445	yes
Serpin H1	P50454	SERPH_HUMAN	46	11.8±5.2	8.7±5.1	-0.44	0.501	
SPARC	P09486	SPRC_HUMAN	35	66.8±15.9	95.5±59	0.52	0.316	
Syndecan-4	P31431	SDC4_HUMAN	22	0.0±0.0	1.1±1.3	n/a	0.186	
Tenascin	P24821	TENA_HUMAN	241	0.0±0.0	3.1±5.9	n/a	0.368	yes
Testican-1	Q08629	TICN1_HUMAN	49	3.5±2.4	5.1±2.1	0.55	0.413	
Thrombospondin-1	P07996	TSP1_HUMAN	129	59.8±30.9	83.8±44.8	0.49	0.336	yes
Thrombospondin-2	P35442	TSP2_HUMAN	130	16.5±5.2	58.0±38.2	1.82	0.148	yes
Transforming growth factor beta-1	P01137	TGFB1_HUMAN	44	0.5±0.9	0.2±0.4	-1.09	0.391	
Transforming growth factor-beta-induced protein ig-h3	Q15582	BGH3_HUMAN	75	47.9±31.4	37.8±16.4	-0.34	0.394	yes
Vasorin	Q6EMK4	VASN_HUMAN	72	0.4±0.9	1.2±1.4	1.44	0.371	
Versican core protein	P13611	CSPG2_HUMAN	265	27.2±8.9	21.1±9.7	-0.36	0.372	yes

Values are averages ± standard deviation of spectral counts. P-values were calculated based on paired t-tests.

Supplementary Table 5: Baseline characteristics of subjects in the Bruneck Study (N=685).

**Characteristics**	**Mean±SD or number(%)**
Age, years	66.1±10.2
Female sex, n (%)	355 (51.8)
Smoking, pack-years	12.7±17.4
Body mass index, kg/m^2	25.4±4.0
Diabetes mellitus, n (%)	79 (11.5)
Hypertension, n (%)	385 (56.2)
HDL-Cholesterol, mg/dL	57.3±15.2
LDL-Cholesterol, mg/dL	148.9±36.7
High-sensitivity CRP, mg/L	3.9±9.5
Atherosclerosis score, mm	3.9±5.5

Supplementary Table 6: Association between markers of plaque vulnerability and incipient and early atherosclerosis in the Bruneck Study (N=670).

	Incipient atherosclerosis				Early atherosclerosis			
	Model 1		Model 2		Model 1		Model 2*	
**Protein**	**Odds Ratio (95% CI)**	**P value**	**Odds Ratio (95% CI)**	**P value**	**Odds Ratio (95% CI)**	**P value**	**Odds Ratio (95% CI)**	**P value**
**FN1**	0.91 (0.72-1.16)	0.451	0.91 (0.71-1.18)	0.48	1.01 (0.84-1.21)	0.935	0.92 (0.75-1.13)	0.432
**CTSD**	1.23 (0.94-1.60)	0.133	1.18 (0.88-1.57)	0.26	1.27 (1.04-1.55)	0.019	1.11 (0.88-1.39)	0.379
**CALP**	0.97 (0.77-1.23)	0.814	0.94 (0.72-1.23)	0.65	1.16 (0.95-1.40)	0.143	0.98 (0.78-1.24)	0.873
**LGALS3BP**	1.32 (0.99-1.77)	0.057	1.25 (0.90-1.72)	0.18	1.38 (1.11-1.71)	0.004	1.13 (0.88-1.45)	0.341
**MMP9**	0.89 (0.68-1.18)	0.419	0.91 (0.68-1.21)	0.52	1.00 (0.82-1.22)	0.991	0.93 (0.74-1.17)	0.567
**CHI3L1**	1.14 (0.84-1.53)	0.411	1.06 (0.76-1.46)	0.75	1.13 (0.91-1.41)	0.282	0.95 (0.74-1.22)	0.668

Odds ratios (OR) are derived from logistic regression analysis and expressed for a 1-SD higher $\log_e$ transformed level of given marker. CI denotes confidence interval. Incipient atherosclerosis refers to the development of first plaques in subjects free of atherosclerosis at baseline.

Model 1 was adjusted for age (years) and sex.

Model 2 was adjusted for age (years), sex, LDL cholesterol (mg/dL), HDL cholesterol (mg/dL), high-sensitivity C-reactive protein (mg/L), diabetes mellitus (0 vs 1), hypertension (0 vs 1), smoking (pack-years), body mass index (kg/m^2).

* Additionally adjusted for atherosclerosis score (mm).

Supplementary Table 7: Association between markers of plaque vulnerability and advanced atherosclerosis (N=670) and cardiovascular disease (N=685) in the Bruneck Study.

	Advanced atherosclerosis				Cardiovascular disease			
	Model 1		Model 2*		Model 1		Model 2	
**Protein**	**Odds Ratio (95% CI)**	**P value**	**Odds Ratio (95% CI)**	**P value**	**Hazard ratio (95% CI)**	**P value**	**Hazard Ratio (95% CI)**	**P value**
**FN1**	1.28(1.01-1.61)	0.038	1.21 (0.93-1.58)	0.163	1.30 (1.05-1.61)	0.015	1.26 (1.01-1.58)	0.040
**CTSD**	1.52 (1.20-1.92)	<0.001	1.58 (1.19-2.11)	0.002	1.38 (1.13-1.68)	0.002	1.30 (1.04-161)	0.019
**CALP**	1.48 (1.15-1.92)	0.003	1.31 (0.93-1.85)	0.125	1.52 (1.21-1.90)	<0.001	1.48 (1.15-1.90)	0.003
**LGALS3BP**	1.68 (1.33-2.12)	<0.001	1.80 (1.33-2.43)	<0.001	1.24 (1.03-1.49)	0.023	1.12 (0.91-1.37)	0.290
**MMP9**	1.45 (1.13-1.87)	0.003	1.53 (1.12-2.08)	0.008	1.40 (1.14-1.73)	0.002	1.36 (1.09-1.69)	0.006
**CHI3L1**	1.40 (1.10-1.78)	0.006	1.48 (1.10-1.99)	0.010	1.31 (1.08-1.59)	0.005	1.32 (1.07-1.63)	0.009

Odds ratios (OR) are derived from logistic regression analysis and hazard ratios (HR) from Cox models. OR and HR are expressed for a 1-SD higher $\log_e$ transformed level of given marker. CI denotes confidence interval.

Model 1 was adjusted for age (years) and sex.

Model 2 was adjusted for age (years), sex, low density lipoprotein cholesterol (mg/dL), high density lipoprotein cholesterol (mg/dL), high-sensitivity C-reactive protein (mg/L), diabetes mellitus (0 vs 1), hypertension (0 vs 1), smoking (pack-years), body mass index (kg/m^2).

* Additionally adjusted for atherosclerosis score (mm).

Supplementary Table 8: Baseline characteristics of cases and age- and sex-matched subcohort members at baseline in the SAPHIR Study.

	Cases (N=58)	Subcohort (N=151)
**Characteristics**	**Mean±SD or number(%)**	**Mean±SD or number(%)**
Age, years	51.9±6.5	51.8±6.3
Female sex, n (%)	14 (24.1)	36 (23.8)
Current smoking, n (%)	13 (22.4)	37 (24.5)
Body mass index, kg/m^2	28.6±5.4	27.0±4.0
Diabetes mellitus, n (%)	2 (3.5)	3 (2.0)
Hypertension, n (%)	47 (81.0)	91 (60.3)
HDL-Cholesterol, mg/dL	51.3±13.8	56.9±15.2
LDL-Cholesterol, mg/dL	155.2±34.2	147.3±33.0
C-reactive protein, mg/L	3.4±4.9	2.5±4.2

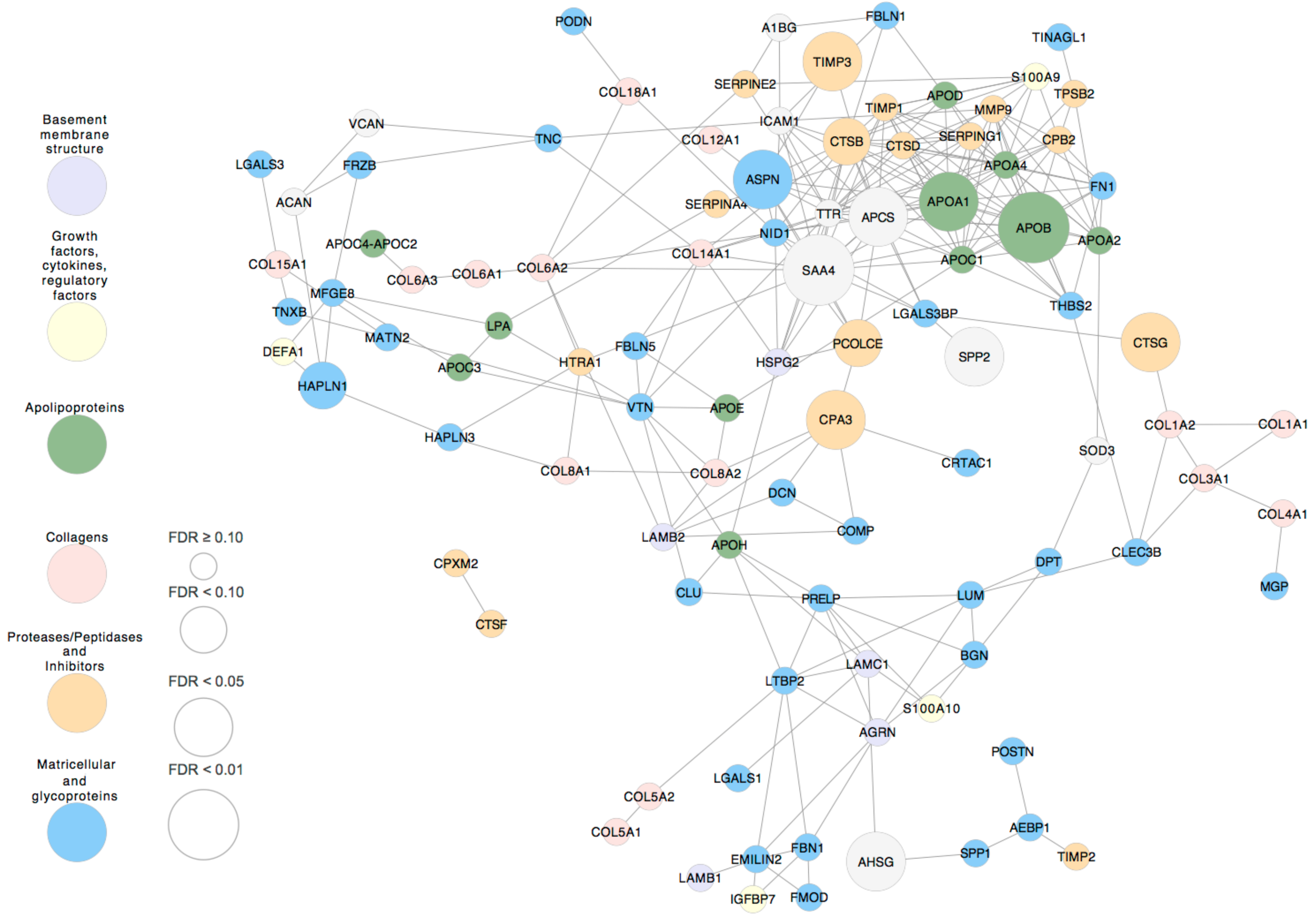

Supplementary Figure 1: Protein network in the GuHCl fraction. Co-expression network in the GuHCl fraction calculated with Pearson correlation values. The edges were correlation values at a FDR<20% level. The sizes of the nodes indicate the level of differential expression between plaques from symptomatic and asymptomatic patients and the colours represent different protein classifications (green - apolipoproteins; pink - collagens; orange - proteases/peptidases and inhibitors; blue – matricellular and glycoproteins; purple - basement proteins; yellow - growth factors, cytokines, and regulatory factors; white - other). The nodes with a thicker grey circle are those which have also been identified at the protein expression level.

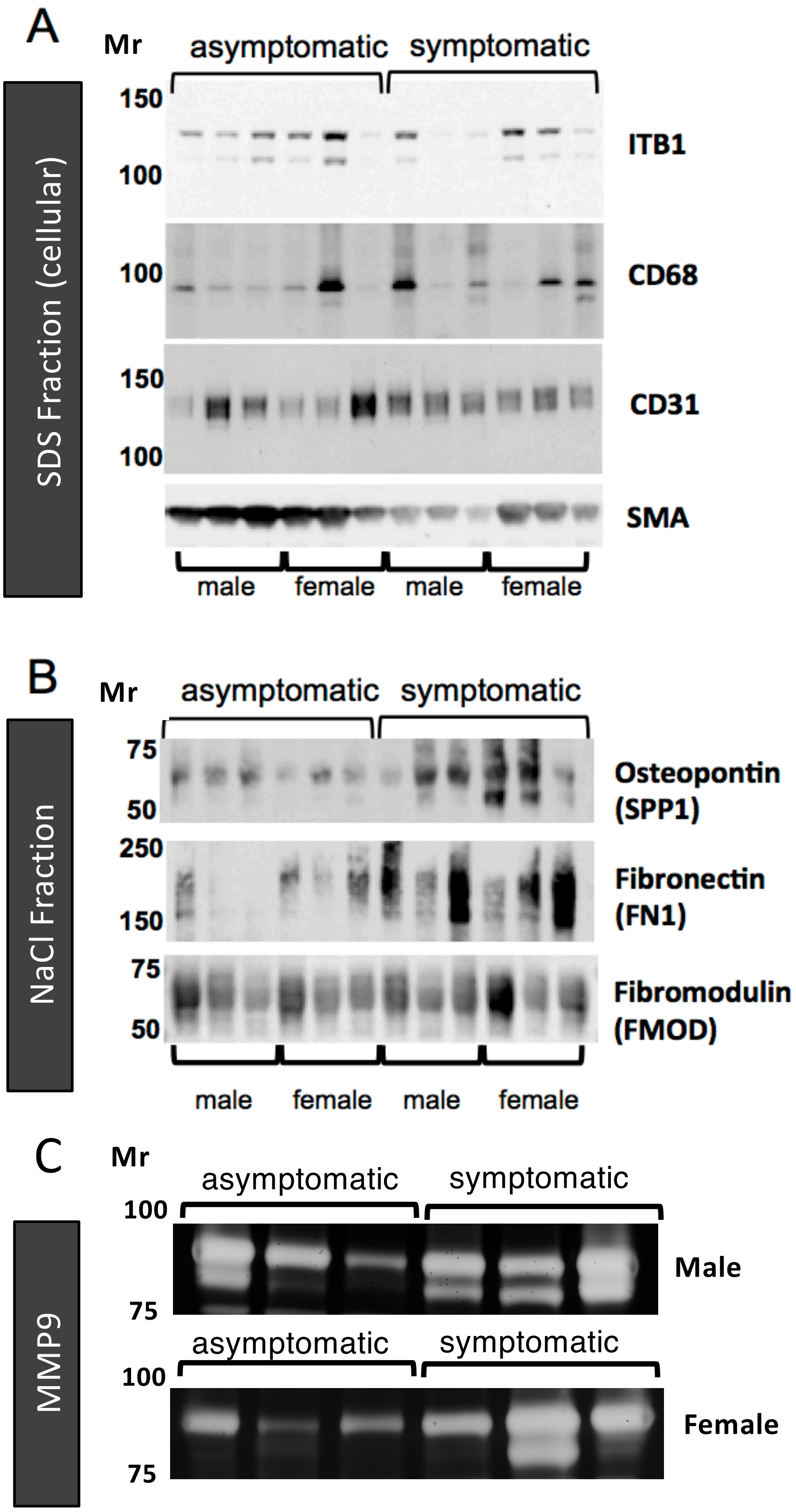

Supplementary Figure 2. Western blots and zymography. (A) First, immunoblotting was performed for selected cellular markers, including integrin beta 1 (ITB1), CD31, CD68, and smooth muscle actin (SMA). Consistent with a stable plaque phenotype, SMA was significantly increased in plaques from asymptomatic patients (p=0.002). CD68 levels, as indicator of the macrophage content, were far more variable between plaques. (B) In agreement with the proteomics data, osteopontin (SPP1) and fibronectin (FN1) were elevated in the NaCl fraction of atherosclerotic plaques from symptomatic patients. SSP1, FN1 And fibromodulin (FMOD) were quantified by immunoblotting. (C) MMP9 in the NaCl fraction was was measured by zymography and, in agreement with the proteomics data, was elevated in the symptomatic patients.

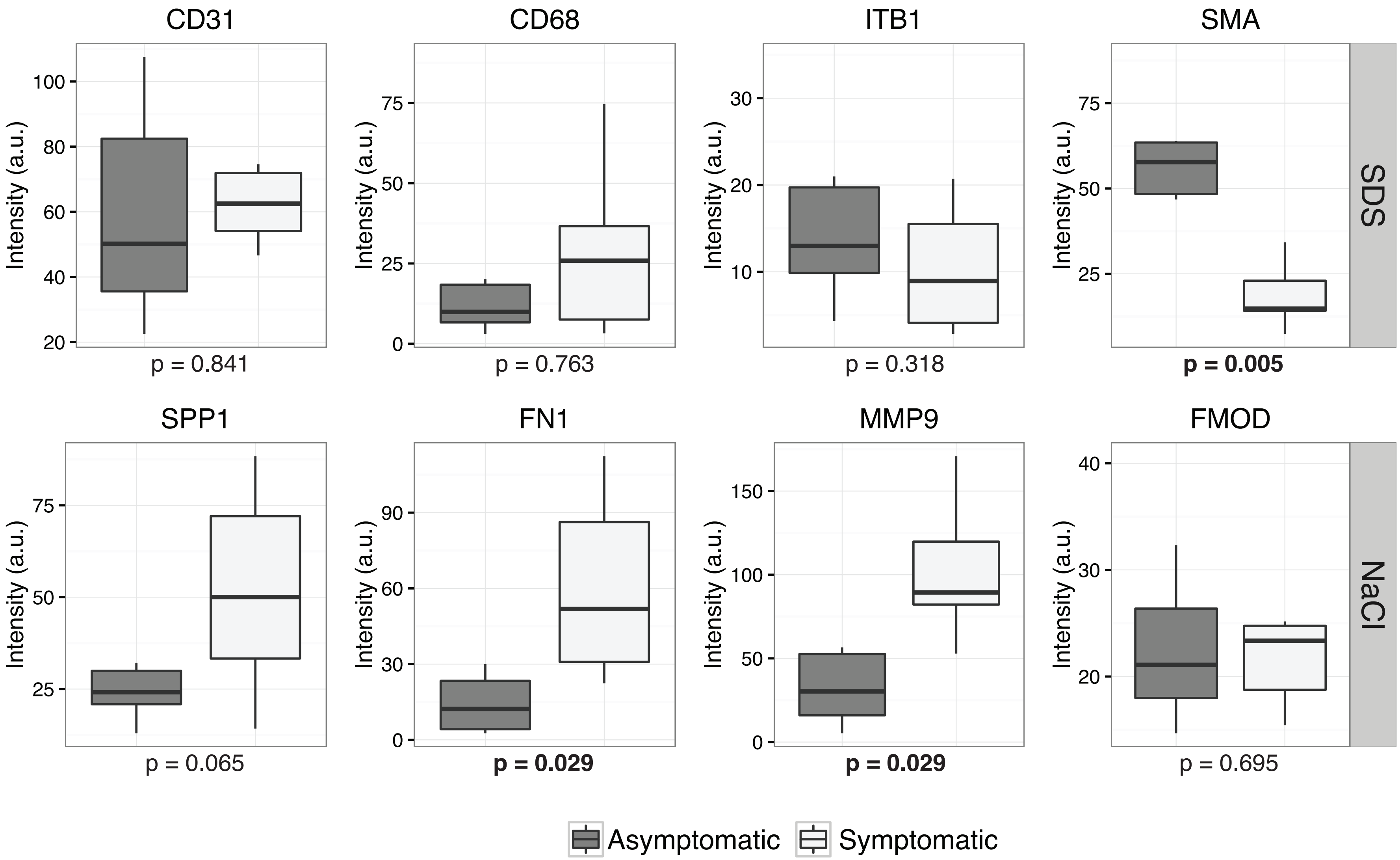

Supplementary Figure 3. Densitometry. ImageJ software (V.1.4.3.67; NIH) was used for the densitometry of each lane of the developed films in Supplementary Figures 2A (upper panel, SDS fraction) and 2B-C (lower panel, NaCl fraction). P-values were derived from unpaired Student's t-test with unequal variance (n=6 per group). ITB1 - Integrin Beta 1; SMA- Smooth Muscle Actin; SPP1 - Osteopontin; FN1 - Fibronectin; MMP9 - Matrix Metallopeptidase 9; FMOD - Fibromodulin.

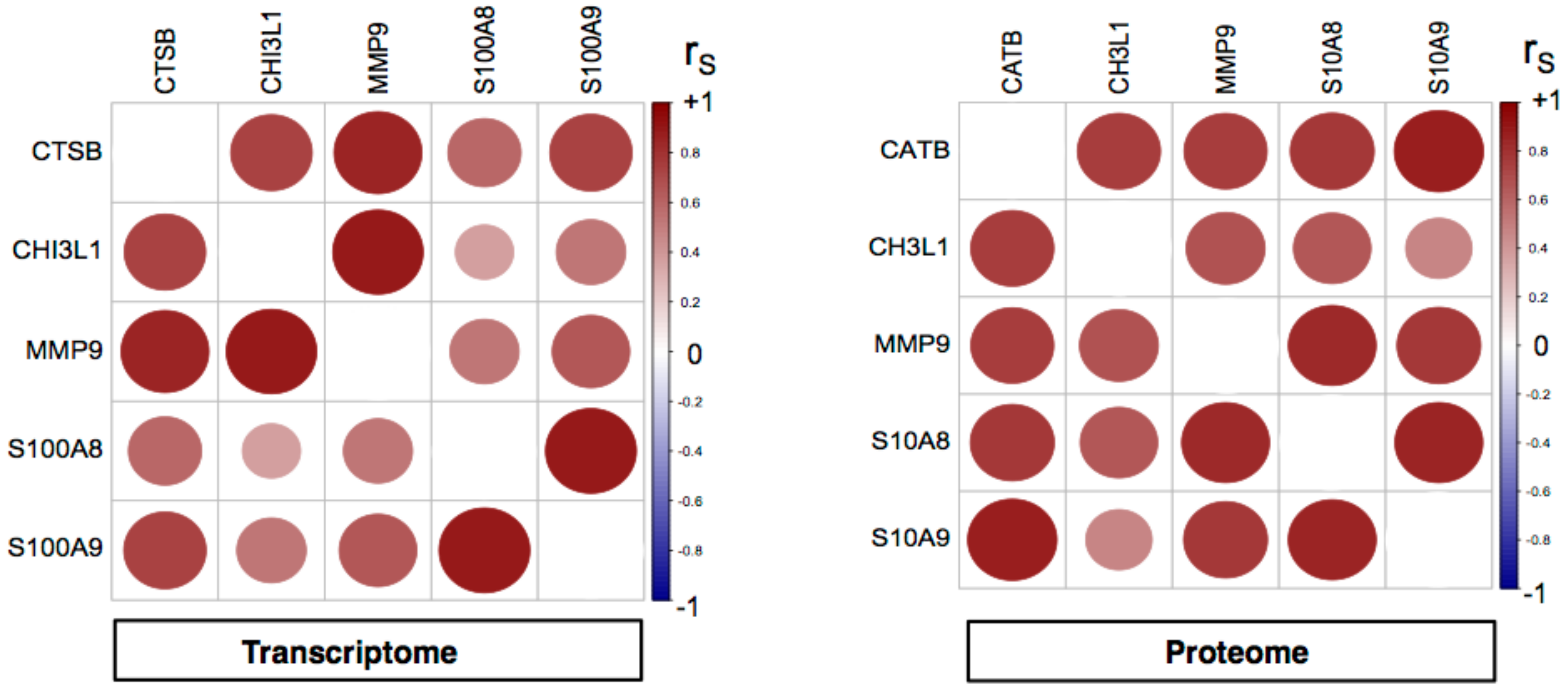

Supplementary Figure 4. Correlations within protein and gene levels. Correlations within the significantly differentially expressed proteins and genes identified 5 in the transcriptomics and proteomics dataset (FDR<10%). The correlation coefficients between the proteins and genes showed a high positive correlation as indicated by the color and size, where the colour is indicated by the scale bar and the size is proportional to the absolute value of the correlation coefficient.

AHSG, Alpha-2-HS-glycoprotein;
APCS, Serum amyloid P-component;
APOA1, Apoliprotein A-I;
APOA2, Apolipoprotein A-II;
APOB, Apolipoprotein B;
APOC3, Apoliprotein C-III;
APOD, Apolipoprotein D;
APOL1, Apoliprotein L1;
APP, Amyloid beta A4 protein;
ASPN, Asporin;
CHI3L1, Chitinase-3-like protein 1;
CLEC3B, Tetranectin;
COL14A1, Collagen alpha-1(XIV) chain;
COL1A1, Collagen alpha-1(I) chain;
COL3A1, Collagen alpha-1(III) chain;
COL6A3, Collagen alpha-3(VI) chain;
COL6A3, Collagen alpha-3(VI) chain;
CPA3, Mast cell carboxypeptidase A;
CRTAC1, Cartilage acidic protein 1;
CTHRC1, Collagen triple helix repeat-containing protein 1;
CTSB, Cathepsin B;
CTSG, Cathepsin G ;
FN1, Fibronectin;
FRZB, Secreted frizzled-related protein 3;
HAPLN1, Hyaluronan and proteoglycan link protein 1;
ICAM1, Intercellular adhesion molecule 1;
LGALS3BP, Galectin-3-binding protein;
LTBP4, Latent-transforming growth factor beta-binding protein 4;
MMP9, Matrix metalloproteinase-9;
OMD, Alpha-1-acid glycoprotein 2;
PCOLCE, Procollagen C-endopeptidase enhancer 1;
PTX3, Pentraxin-related protein PTX3;
S100A8, Protein S100-A8;
S100A9, Protein S100-A9;
SAA4, Serum amyloid A-4 protein;
SERPINA4, Kallistatin;
SPP1, Osteopontin;
SPP2, Secreted phosphoprotein 24;
TIMP1, Metalloproteinase inhibitor 1;
TIMP2, Metalloproteinase inhibitor 2;
TIMP3, Metalloproteinase inhibitor 3;
TNC, Tenascin.

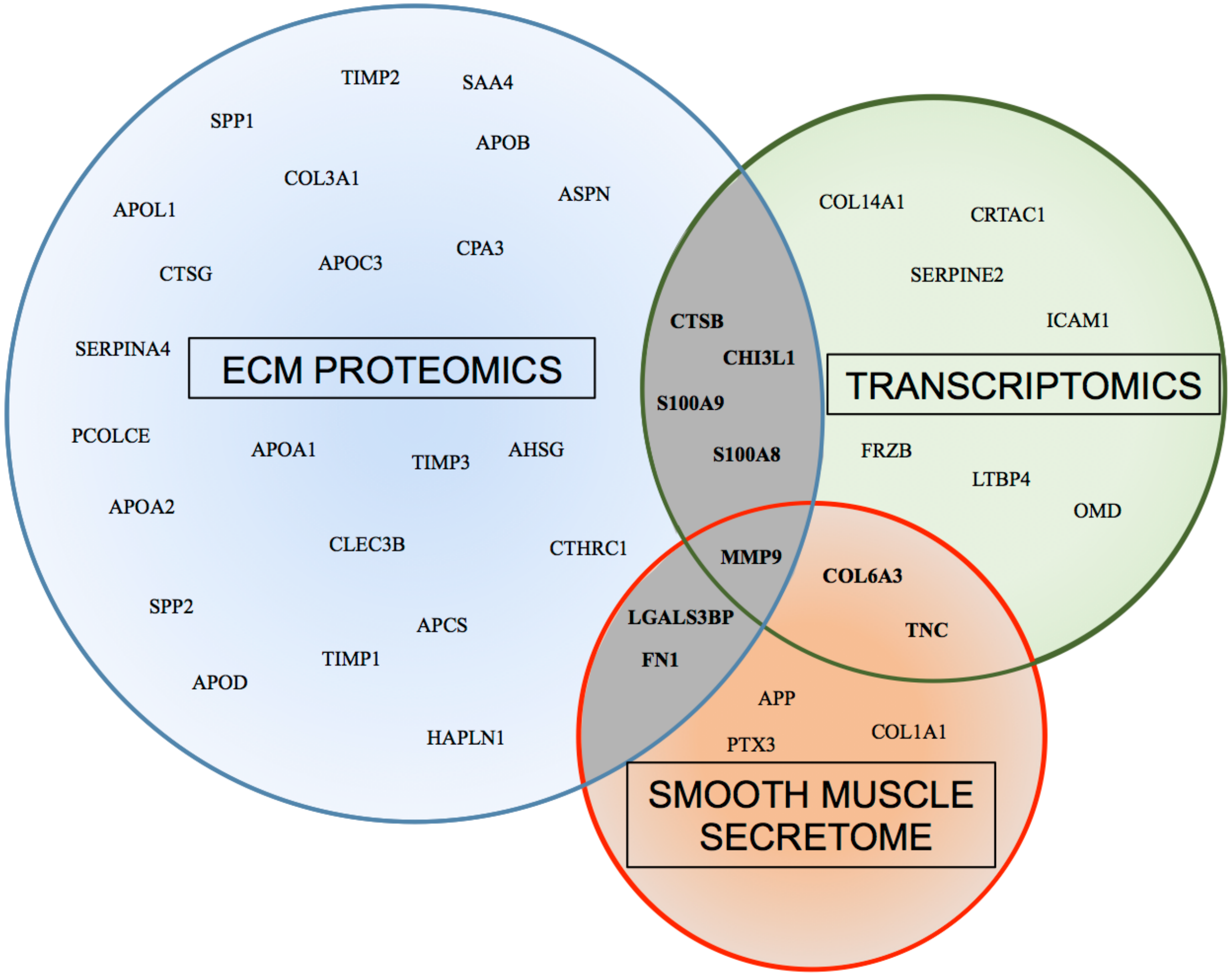

Supplementary Figure 5: Venn diagram. Proteins differentially expressed in plaques from symptomatic versus asymptomatic patients using ECM proteomics (blue circle) or ECM transcriptomics (green circle), as well as proteins found in the secretome (deposited ECM and conditioned media) of lipid-loaded versus unstimulated SMCs (red circle), and their overlap (grey area). Candidate proteins required identification by ECM proteomics and at least one of the other two discovery approaches.

Marker / Model	HR (95% CI) per SD	P Value
**CTSD**		
M1	1.46 (1.17, 1.83)	0.001
M2	1.32 (1.05, 1.68)	0.020
**CALP**		
M1	1.28 (1.03, 1.60)	0.024
M2	1.24 (0.97, 1.58)	0.093
**LGALS3BP**		
M1	1.43 (1.07, 1.92)	0.015
M2	1.35 (1.01, 1.79)	0.042
**MMP-9**		
M1	1.51 (1.17, 1.94)	0.001
M2	1.63 (1.26, 2.11)	<0.001
**CHI3L1**		
M1	0.92 (0.71, 1.20)	0.534
M2	0.91 (0.71, 1.18)	0.490

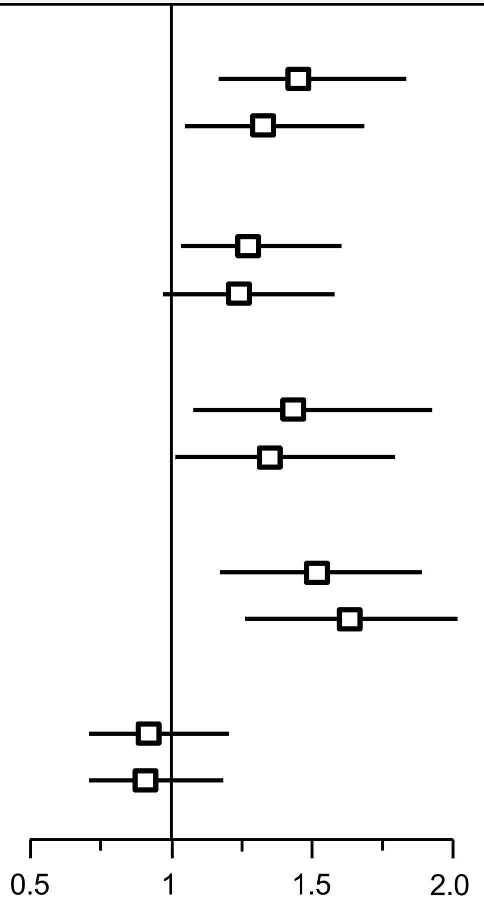

Supplementary Figure 6: Association between markers of plaque vulnerability and CVD in the Saphir Study. Hazard ratios were derived from Cox proportional hazard regression models using time-on-study as a timescale and applying Prentice weights and robust SEs (case-cohort design). HRs were expressed for a 1-SD higher $\log_e$-transformed level of given marker. Model 1 (M1) was adjusted for age and sex. Model 2 (M2) was adjusted for age, sex, LDL and HDL cholesterol, high-sensitivity C-reactive protein, diabetes mellitus, hypertension, smoking, and body mass index. CTSD, cathepsin D; CALP, calprotectin; LGALS3BP, galectin-3-binding protein; MMP9, matrix metalloproteinase-9; CHI3L1, chitinase-3-like protein 1.

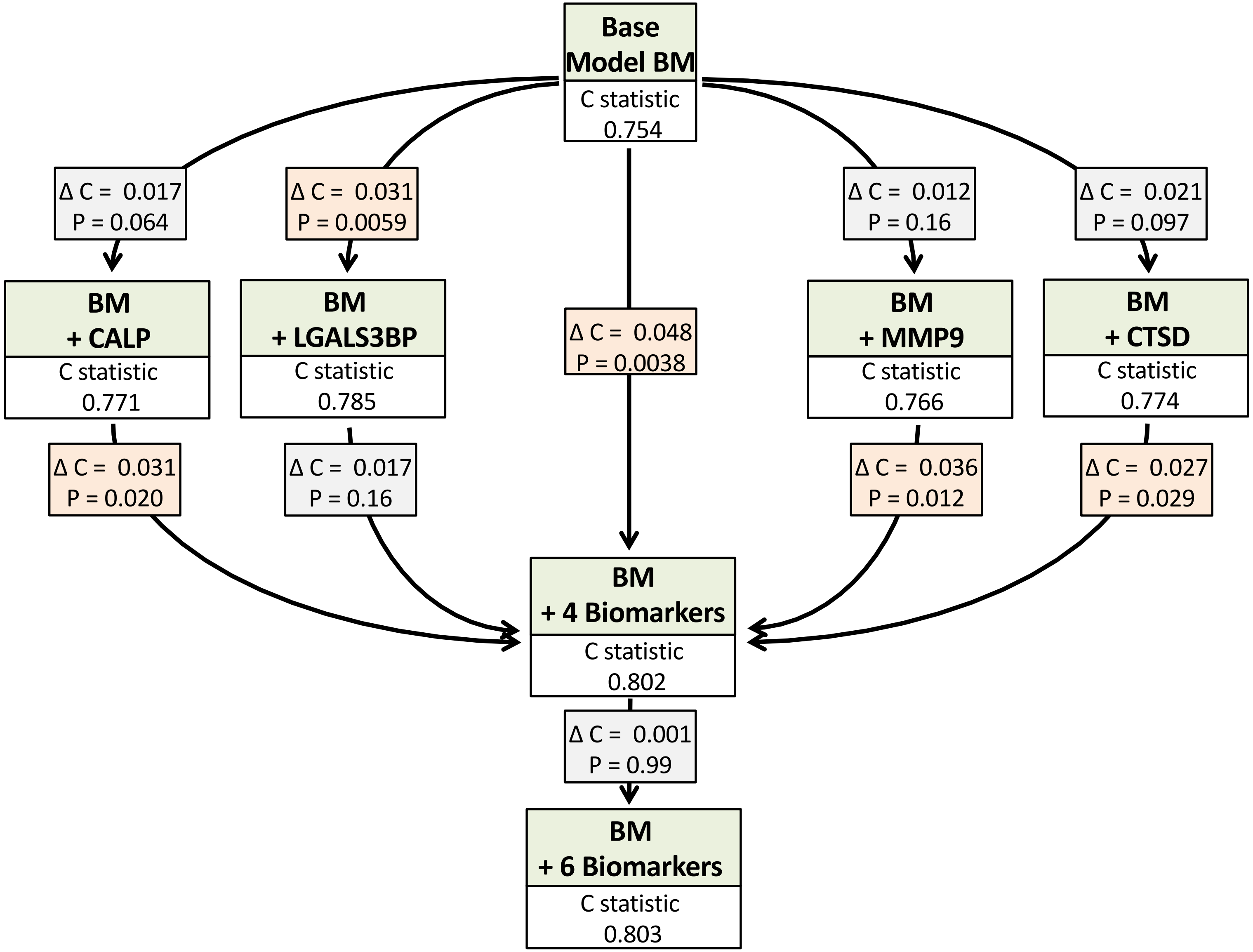

Supplementary Figure 7: Improvement in discrimination of the risk of advanced atherosclerosis (progression 2000 to 2010) with the use of single and combined markers of symptomatic plaques (Bruneck Study). The C-statistic, a measure of risk discrimination, was calculated as the area under a receiver operating characteristic (ROC) curve. The base model (BM) was adjusted for age, sex, LDL and HDL cholesterol, diabetes mellitus, hypertension, and smoking. Changes in the C-statistic (Δ C) and corresponding P values are presented for addition of the individual markers of plaque vulnerability to the base model (CALP, LGALS3BP, MMP, CTSD) and for addition of the entire 4-biomarker signature as well as a 6-biomarker signature also considering FN1 and CHI3L1. CALP, calprotectin; LGALS3BP, galectin-3-binding protein; MMP9, matrix metalloproteinase-9; CTSD, cathepsin D; FN1, fibronectin; CHI3L1, chitinase-3-like protein 1.

Cohorts	Bruneck Study	Saphir Study
Outcome	CVD	CVD
Model	Base Model<br>+ 4 Biomarkers	Base Model<br>+ 4 Biomarkers
Discrimination	Δ C = 0.029<br>P = 0.030	Δ C = 0.066<br>P = 0.039
Reclassification	Cont. NRI = 0.358 (0.134 to 0.582)<br>P = 0.002<br><br>IDI = 0.040 (0.016 to 0.063)<br>P = 0.001<br><br>NRI = 0.104 (0.031 to 0.177)<br>P = 0.005	Cont. NRI = 0.461 (0.146 to 0.777)<br>P = 0.004<br><br>IDI = 0.036 (0.012 to 0.059)<br>P = 0.003<br><br>NRI = 0.159 (-0.018 to 0.336)<br>P = 0.078

Supplementary Figure 8: Incremental predictive value of the 4-biomarker signature consisting of CALP, LGALS3BP, MMP, and CTSD in the Bruneck and Saphir Studies. The base model (BM) was adjusted for age, sex, LDL and HDL cholesterol, diabetes mellitus, hypertension, and smoking. The C-statistic (logistic regression models) and C-index (Cox models) and changes thereof upon addition of the 4-biomarker signature were calculated as measures of risk discrimination while the continuous net reclassification index (Cont. NRI), the integrated discrimination improvement (IDI) and the categorical retrospective NRI across predicted 10 year CVD risk categories (<5%, 5% to <7.5%, and ≥7.5%) were calculated as measures of risk reclassification. CALP, calprotectin; LGALS3BP, galectin-3-binding protein; MMP9, matrix metalloproteinase-9; CTSD, cathepsin D.

Supplementary Figure 9. Carotid ultrasound protocol of the Bruneck Study. ICA denotes internal carotid artery; CCA, common carotid artery .

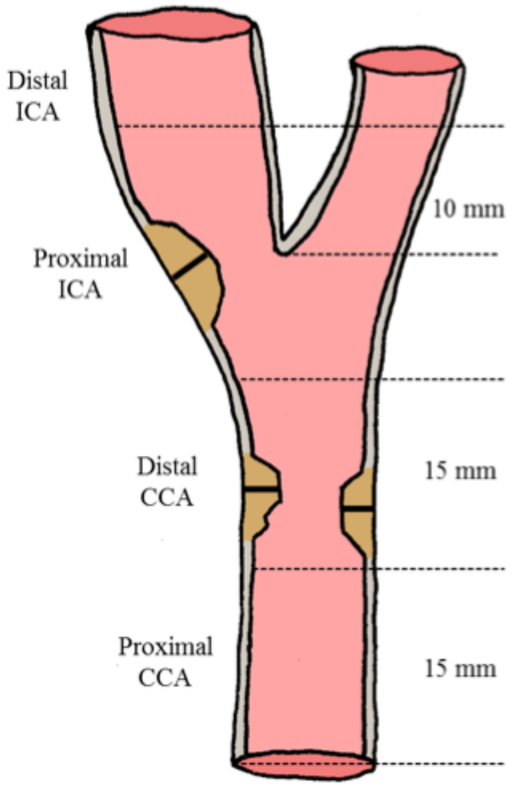

Supplementary Figure 10. Person-based progression model of atherosclerosis in the Bruneck Study. Pre-existing atherosclerotic plaques may experience one of two types of disease progression: a) early atherogenesis or b) advanced atherogenesis.

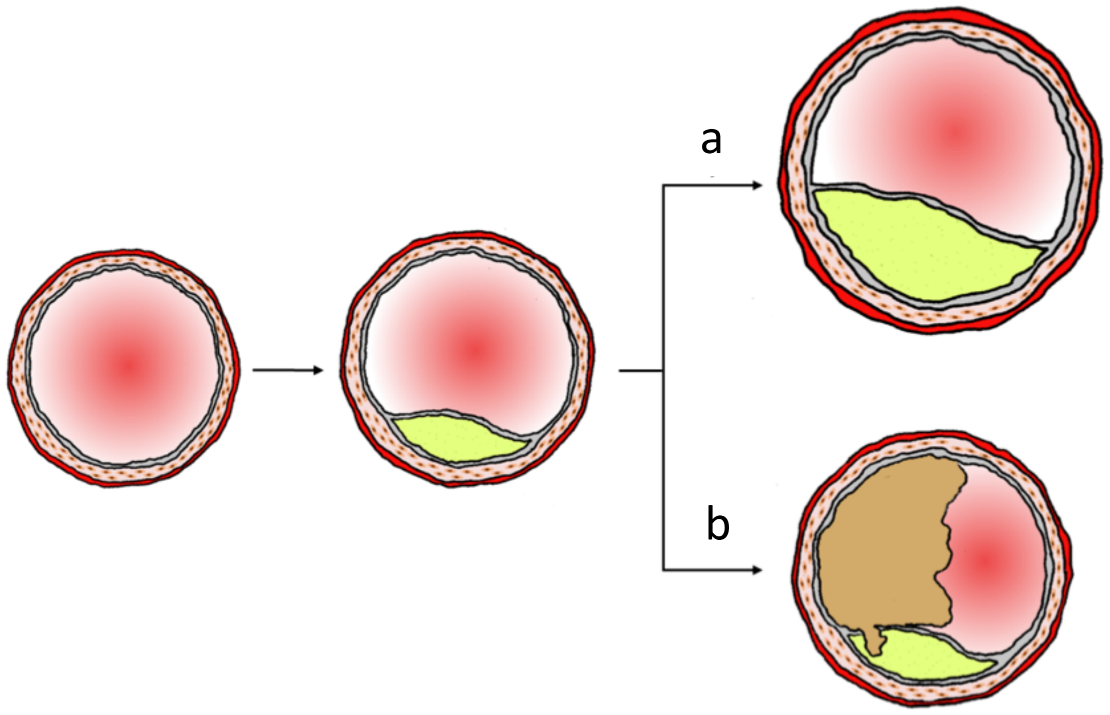

Supplement: Supplemental data [file jci-127-86924-s001.pdf]
